# SHP2 Inhibition with TNO155 Increases Efficacy and Overcomes Resistance of ALK Inhibitors in Neuroblastoma

**DOI:** 10.1158/2767-9764.CRC-23-0234

**Published:** 2023-12-27

**Authors:** Ivette Valencia-Sama, Lynn Kee, Gabriella Christopher, Michael Ohh, Mehdi Layeghifard, Adam Shlien, Madeline N. Hayes, Meredith S. Irwin

**Affiliations:** 1 Cell Biology Program, The Hospital for Sick Children, Toronto, Canada.; 2 Department of Laboratory Medicine and Pathobiology, University of Toronto, Toronto, Canada.; 3 Department of Biochemistry, University of Toronto, Toronto, Canada.; 4 Genetics and Genomics Program, The Hospital for Sick Children, Toronto, Canada.; 5 Developmental and Stem Cell Biology Program, The Hospital for Sick Children, Toronto, Canada.; 6 Department of Molecular Genetics, University of Toronto, Toronto, Canada.; 7 Department of Medical Biophysics, University of Toronto, Toronto, Canada.; 8 Department of Paediatrics, The Hospital for Sick Children, Toronto, Canada.

## Abstract

Survival rates among patients with high-risk neuroblastoma remain low and novel therapies for recurrent neuroblastomas are required. *ALK* is commonly mutated in primary and relapsed neuroblastoma tumors and ALK tyrosine kinase inhibitors (TKI) are promising treatments for ALK-driven neuroblastoma; however, innate or adaptive resistance to single-agent ALK-TKIs remain a clinical challenge. Recently, SHP2 inhibitors have been shown to overcome ALK-TKI resistance in lung tumors harboring *ALK* rearrangements. Here, we have assessed the efficacy of the SHP2 inhibitor TNO155 alone and in combination with the ALK-TKIs crizotinib, ceritinib, or lorlatinib for the treatment of ALK-driven neuroblastoma using *in vitro* and *in vivo* models. In comparison to wild-type, ALK-mutant neuroblastoma cell lines were more sensitive to SHP2 inhibition with TNO155. Moreover, treatment with TNO155 and ALK-TKIs synergistically reduced cell growth and promoted inactivation of ALK and MAPK signaling in ALK-mutant neuroblastoma cells. ALK-mutant cells engrafted into larval zebrafish and treated with single agents or dual SHP2/ALK inhibitors showed reduced growth and invasion. In murine ALK-mutant xenografts, tumor growth was likewise reduced or delayed, and survival was prolonged upon combinatorial treatment of TNO155 and lorlatinib. Finally, we show that lorlatinib-resistant ALK-F1174L neuroblastoma cells harbor additional RAS-MAPK pathway alterations and can be resensitized to lorlatinib when combined with TNO155 *in vitro* and *in vivo*. Our results report the first evaluation of TNO155 in neuroblastoma and suggest that combinatorial inhibition of ALK and SHP2 could be a novel approach to treating ALK-driven neuroblastoma, potentially including the increasingly common tumors that have developed resistance to ALK-TKIs.

**Significance:**

These findings highlight the translatability between zebrafish and murine models, provide evidence of aberrant RAS-MAPK signaling as an adaptive mechanism of resistance to lorlatinib, and demonstrate the clinical potential for SHP2/ALK inhibitor combinations for the treatment of ALK-mutant neuroblastoma, including those with acquired tolerance or potentially resistance to ALK-TKIs.

## Introduction

Neuroblastoma, the most common extracranial solid tumor in children, accounts for approximately 15% of pediatric cancer–related mortality. While 90% of patients with neuroblastoma with low and intermediate risk have excellent outcomes, only approximately 50% of high-risk patients are cured with intensive multimodal therapies (1). Metastasis and therapy resistance remain major challenges for patients with relapsed high-risk neuroblastoma, and more effective single-agent and combination therapies to treat disease recurrence are currently lacking.

The anaplastic lymphoma kinase (*ALK*) gene encodes a receptor tyrosine kinase that is commonly hyperactivated in several cancers through chromosomal translocations or activating point mutations (2–4). In neuroblastoma at diagnosis, *ALK* mutations or amplifications are detected in 14% of high-risk tumors and their presence is associated with unfavorable outcomes (5–8). Moreover, next-generation sequencing studies have identified a high frequency of *ALK* aberrations, including clonally selected or *de novo ALK* mutations in relapsed neuroblastomas (9–11). These studies highlight *ALK* as a common driver of neuroblastoma at diagnosis and recurrence, and a tractable therapeutic candidate.

Although several ALK tyrosine kinase inhibitors (TKI) have been evaluated, to date none have been approved for clinical use in patients with neuroblastoma. Targeting neuroblastoma with single-agent ALK-TKIs has shown efficacy in subsets of patients; however, tumor resistance commonly occurs. Crizotinib, a first-generation ALK inhibitor approved for *ALK*-translocated lung tumors, was less effective in preclinical neuroblastoma models, especially in tumors harboring the neuroblastoma-associated ALK-F1174L mutation which showed innate resistance to this therapy (12–14). Its successor, ceritinib, which overcame crizotinib resistance in a subset of lung cancers (15), showed moderate responses in preclinical ALK-driven neuroblastoma models (16); however, only 20% of patients demonstrated neuroblastoma tumor response, followed in many by subsequent progression, likely due to adaptive mechanisms of resistance (16, 17). The third-generation inhibitor, lorlatinib, has shown more promising activity, including in ALK-TKI–resistant neuroblastoma tumors (18–20); however, some patients eventually recur, and sequencing of serial circulating tumor DNA (ctDNA) samples obtained during treatment has shown the emergence of resistance mutations, including many alterations predicted to activate the RAS-MAPK pathway (21–23). Furthermore, recent studies using unbiased proteomic approaches and CRISPR screens have revealed several important mechanisms of resistance following lorlatinib treatment of different ALK-driven tumors, including alterations in RAS/MAPK and PI3K/AKT/mTOR signaling (24–27).

The protein tyrosine phosphatase SHP2 (encoded by *PTPN11*) mediates the activation of the MAPK and PI3K signaling cascades, and pharmacologic inhibition or depletion of SHP2 suppresses tumor growth in several cancer models, including neuroblastoma (28–30). RAS-MAPK activation has been consistently associated with ALK hyperactivation and/or adaptive resistance to ALK inhibitors (11, 24, 31, 32), and multiple combination strategies have been proposed to increase drug efficacy. We previously reported that combinations of SHP2 inhibitors with other RAS pathway inhibitors, such as trametinib and ulixertinib, were synergistic in neuroblastoma *in vitro* and *in vivo* (30). Interestingly, dual inhibition of SHP2 and ALK has been shown to be an effective approach for *ALK*-translocated lung tumors and lymphomas that are resistant to ALK inhibitors (33–35), suggesting potential benefits of combining ALK and SHP2 inhibitors. Furthermore, phosphoproteomics and proximitome analyses of ALK in neuroblastoma cells have revealed SHP2 as a downstream target and interactor of active ALK (31, 36). However, the feasibility and efficacy of ALK and SHP2 inhibitor combinations for neuroblastoma has not been assessed.

Here, we evaluate single-agent and combination activity of the SHP2 inhibitor TNO155 (37) using *in vitro* and *in vivo* models of ALK-driven neuroblastoma. Our results show that combining TNO155 with ALK inhibitors currently in clinical use reduces tumor growth in neuroblastoma cell lines, zebrafish, and murine models harboring *ALK* mutations. Our studies provide the first evidence for neuroblastoma sensitivity to the SHP2 inhibitor TNO155 and its efficacy in combination with ALK inhibitors.

## Materials and Methods

### Cell Lines

Neuroblastoma cell lines SK-N-SH, LAN-6, LAN-5, Kelly, NB1643, SH-SY5Y, IMR-32, NGP, CHP-21, SK-N-AS, COG-N-415, and COG-N-519 were purchased from ATCC or obtained from COG Childhood Cancer Repository (http://cccells.org). Cell culture conditions are described in Supplementary Materials and Methods. Prior to experimental use, cells were tested for *Mycoplasma* (InvivoGen). Cell authentication using short tandem repeat analysis was performed at The Centre for Applied Genomics (TCAG) Sequencing Facility at the Hospital for Sick Children (Toronto, Ontario, Canada).

### Chemicals

TNO155 (#HY-136173) and SHP099 (#HY-100388A) were purchased from MedChemExpress, and ceritinib (#S7083), lorlatinib (#S7536), and crizotinib (#S1068) were purchased from Selleck Chemicals. Inhibitors were resuspended in DMSO. For murine experiments, lorlatinib (#HY-12215) and TNO155 (#HY-136173) were purchased from MedChemExpress or TNO155 was provided by Novartis AG. Drugs were resuspended in final concentrations of 0.5% methylcellulose + 0.5% Tween 80 in water.

### Cell Viability Assays

A total of 3–5 × 10^3^ cells per well were treated with inhibitors in 96-well plates for 72 hours. AlamarBlue reagent (Invitrogen) was added overnight, and fluorescence was assessed using a microplate reader (Spectra MAX Gemini EM, Molecular Devices) with a l540 excitation/l590 emission filter provided by the Imaging Facility at The Hospital for Sick Children (Toronto, Ontario, Canada). IC_50_ curves were calculated using GraphPad Prism 9 software (GraphPad Software Inc.). For each condition, experiments included 3–6 technical replicates and were performed ≥ 3 times.

### RAS-GTP Pulldowns and Immunoblotting

For RAS-GTP pulldowns, Kelly-S and Kelly-LR cells were lysed and 2 mg of whole cell extraction (WCE) lysates were precipitated using immobilized GTP beads (Jena Bioscience) as described previously (30). For immunoblotting analyses, cells were lysed and protein lysates were quantified, resolved, electrotransferred, and probed with the indicated antibodies as described previously (30). List of antibodies is included in Supplementary Materials and Methods.

### Statistical and Synergy Analyses

Unpaired two-tailed Student *t* test was used to assess statistical significance between two treatment groups. ANOVA followed by a *post hoc* Tukey test was used for multiple group comparisons. For Kaplan–Meier survival curves, log-rank (Mantel–Cox) tests were performed. Excess over Bliss (EOB) assessments were calculated as described previously (38) with EOB values > 0 considered synergistic responses. Data reported as mean and SD represents three independently conducted experiments. Statistical analyses were performed using GraphPad Prism 9 (GraphPad Software Inc.), and *P* value < 0.05 were considered significant.

### Animal Studies

For zebrafish larval xenografts, at 2 days post fertilization (dpf) Casper strain embryos were anesthetized in 200 mg/L buffered Tricaine, laterally immobilized in 3% methylcellulose, and microinjected into the yolk sac with 100–120 GFP-expressing cells (pcDNA3.1 vector backbone) costained with 1,1′-dioctadecyl-3,3,3′,3′-tetramethylindocarbocyanine perchlorate (DiI). The following day (3 dpf), embryos were screened for engraftment and aliquoted into 24-well plates containing TNO155 (5 or 10 µmol/L), ceritinib (1 or 1.5 µmol/L), lorlatinib (3 or 5 µmol/L) or combination treatments diluted in zebrafish egg water (60 µg/mL sea salt in RO water + 0.1% methylene blue). Engrafted embryos were submersed in drug and incubated at 35°C for 72 hours. At 72 hours posttreatment (6 dpf), tumor mass was assessed using an Axiozoom.V16 fluorescent macroscope (Zeiss) or a Nikon AR1 confocal microscope. Images captured using ZEN 2.5 Pro software were quantified using GFP fluorescence area × pixel intensity (ImageJ software), normalized to pretreatment, and expressed as relative growth.

For mouse xenografts, female NOD/SCID mice (6–8 weeks) were injected subcutaneously with 5 × 10^6^ Kelly cells in 0.1 mL suspension containing 50% Matrigel (Corning) in PBS. Once tumors reached 60–100 mm^3^, mice were randomized to receive a 0.2 mL suspension containing either vehicle, TNO155 (7.5 or 10 mg/kg twice per day), lorlatinib (1 or 1.5 mg/kg twice per day), or the combination by oral gavage 5 days per week, similar to other treatment regimens (39). Tumor volumes were monitored at least twice per week, and endpoint was defined as measurements over 10 mm in two of three volume dimensions. All animal studies were performed in accordance with University Health Network and SickKids Institutional Animal Utilization Protocol guidelines.

### Whole-genome Sequencing Analysis

Whole-genome sequencing (WGS) of Kelly-S and Kelly-LR samples were performed by TCAG Next Generation Sequencing Facility at the Hospital for Sick Children (Toronto, Ontario, Canada) using an Illumina NovaSeq 16000. The sequencing depth was 102X for the Kelly-LR sample and 132X for the Kelly-S sample. FASTQ files were aligned using BWA-MEM (v0.78) and PCR duplicates were marked with Sambamba (v0.7.0), with indel realignment (GATK v3.8) and base quality recalibration using GATK (v4.1.3). Somatic substitution calling was performed with Mutect2 (GATK v4.1.3) in a matched tumor-normal pair approach. To call somatic mutations exclusive to the Kelly-LR sample, the Kelly-S sample was used as a matched normal. In addition, Strelka (v2.9.10) and MuSE (v2.0) were also used to call somatic mutation in the exact same matched tumor-normal pair approach. Variants detected by all three mutation callers were selected as the final consensus set of variants.

### Mass Spectrometry and Phosphoproteomics Analyses

All sample preparation, LC/MS-MS data collection, and mass spectrometry (MS) data searches were carried out at SPARC BioCentre at The Hospital for Sick Children (Toronto, Ontario, Canada).

Samples were digested with trypsin, enriched for phosphorylated serine, threonine and tyrosine (pSTY) sites with Fe-NTA beads, precipitated, and resuspended in 2% acetonitrile (ACN) + 0.1% formic acid. Peptides were dried by vacuum centrifugation, resuspended in buffer A, and analyzed by LC/MS-MS using an Evosep One LC system 15 SPD Method and an Orbitrap Fusion Lumos Tribrid Mass Spectrometer (Thermo Fisher Scientific). MS raw files were analyzed using PEAKS Studio software (Bioinformatics Solutions Inc.) and Proteome Discoverer (version 2.5.0.400), and fragment lists searched against the human UniProt Reference database (Uniprot_UP000005640_Human_15092020.fasta). Additional information is available in Supplementary Materials and Methods.

### Data Availability

All data generated are available upon request from the corresponding author.

## Results

### ALK-mutant Neuroblastoma Cells are Sensitive to SHP2 Inhibitors

**FIGURE 1:**
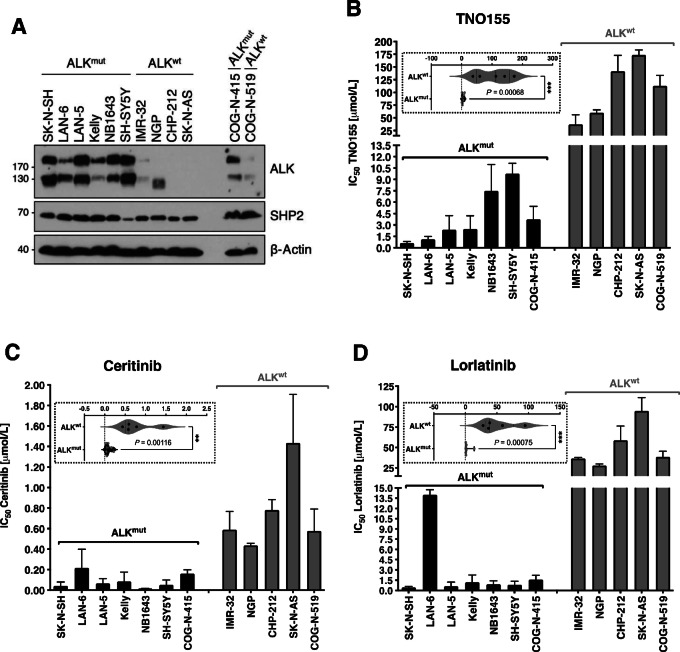
ALK-mutant neuroblastoma cell lines are sensitive to SHP2 inhibition. **A,** Protein expression in a panel of neuroblastoma cell lines with ALK-mutant (ALK^mut^) or wild-type (ALK^wt^) status. Calculation of IC_50_ in neuroblastoma cells following treatment with TNO155 (**B**), ceritinib (**C**), or lorlatinib (**D**) for 72 hours. **, *P* < 0.01; ***, *P* < 0.001.

A panel of 12 neuroblastoma cell lines with *ALK* mutant (ALK^mut^) or wild-type (ALK^wt^) status were selected to assess sensitivity to SHP2 inhibition (Table 1). In line with previous reports (40), ALK expression was generally increased in cells harboring *ALK* mutations, whereas SHP2 expression was similar among most cells regardless of *ALK* status (Fig. 1A; Supplementary Fig. S1A–S1D). IC_50_ were determined after treating cells with increasing concentrations of the allosteric SHP2 inhibitor TNO155. Similar to our published data for SHP099 (ref. 30; Supplementary Fig. S1E), ALK^mut^ cell lines were more sensitive to SHP2 inhibition with TNO155, with average IC_50_ values significantly lower than ALK^wt^ cells (Fig. 1B). Notably, SH-SY5Y cells, which have relatively lower SHP2 expression, were the least sensitive to TNO155 among the ALK^mut^ cells. Moreover, consistent with previous reports, cell lines bearing *NRAS* mutations (CHP-212 and SK-N-AS) were the most insensitive to TNO155 (30, 41). As expected, ALK^mut^ cells were more sensitive to all ALK-TKIs tested including crizotinib, ceritinib, and lorlatinib (Supplementary Fig. S2A; Fig. 1C and D). Of note, LAN-6 cells, which harbor an *ALK* mutation outside of the tyrosine kinase domain, were relatively less sensitive to ALK-TKIs; however, consistent with other reports assessing SHP099 (41), sensitivity to TNO155 was likely observed because of LAN-6 cells also expressing a KRAS-G12C mutation, which preserves high intrinsic GTPase activity (42). Overall, the average IC_50_ of ALK^mut^ cells treated with TNO155, ceritinib or lorlatinib was approximately 26-fold, approximately 9-fold, and approximately 18-fold lower than ALK^wt^ cells, respectively (Table 1). Taken together, this suggests that SHP2 inhibitors, like ALK-TKIs, are more effective in the context of ALK hyperactivation and that this selectivity could be exploited in ALK-mutant neuroblastoma.

**TABLE 1:** Mutational status and IC_50_ values of neuroblastoma cell lines

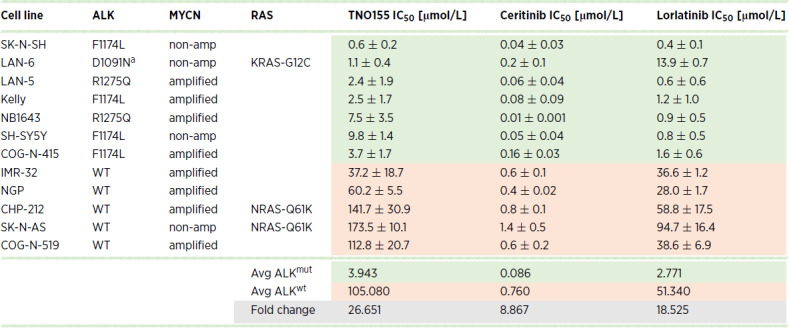						

Abbreviations: amp, amplified; avg, average; mut, mutation; wt, wild-type.

^a^Mutation not within tyrosine kinase domain.

^±^Standard deviation.

### TNO155 has Synergistic Activity with ALK-TKIs in Neuroblastoma Cells

**FIGURE 2:**
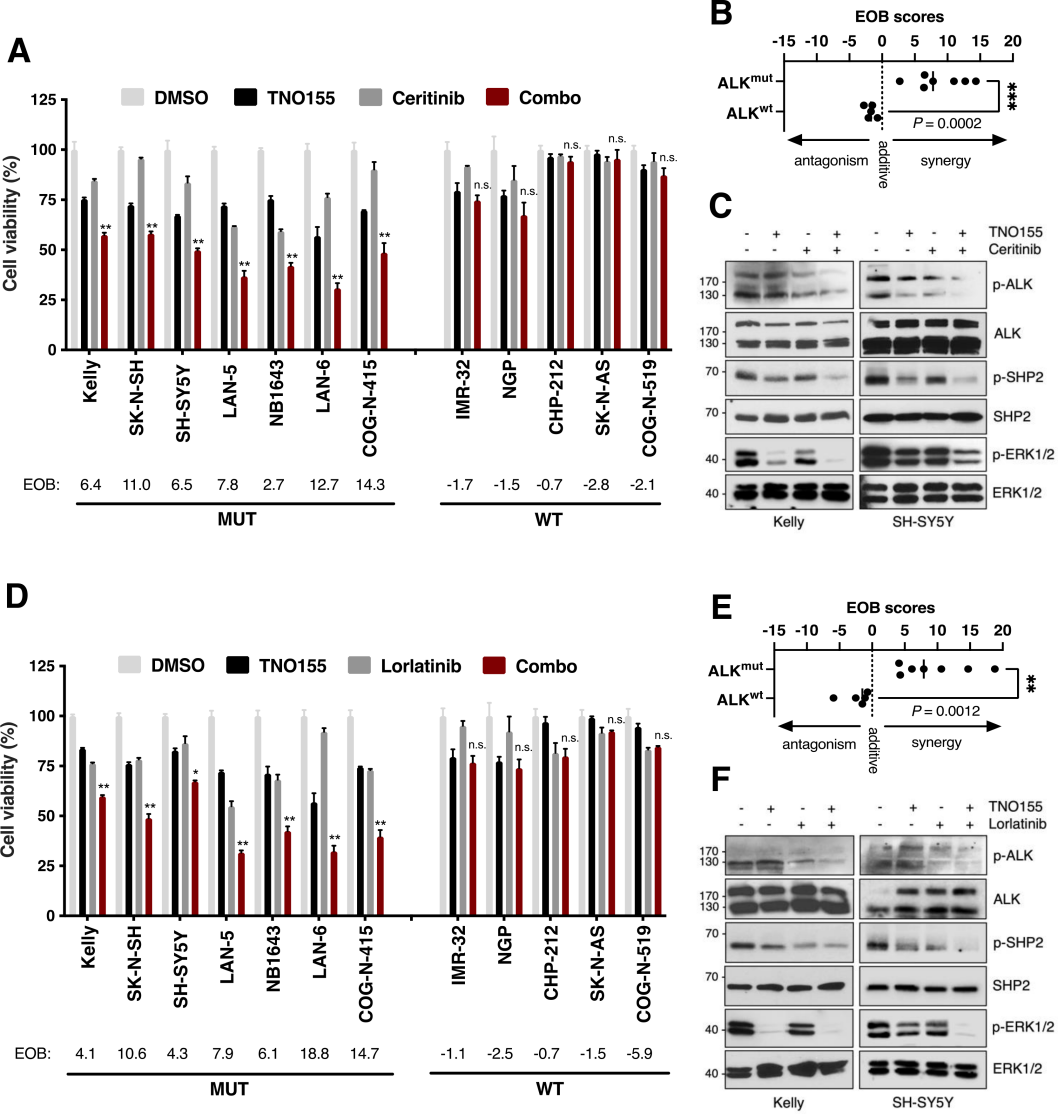
TNO155 synergizes with ALK-TKIs in ALK-mutant neuroblastoma cells. Cell viability (alamarBlue) analysis (**A**) and determination of drug interaction (**B**) in neuroblastoma cells with ALK wild-type (WT) or mutant (MUT) status treated with DMSO control, TNO155, ceritinib, or TNO155 plus ceritinib for 72 hours. **C,** Western immunoblots of Kelly and SH-SY5Y cells treated with TNO155 (1 µmol/L), ceritinib (0.1 µmol/L), or combination for 24 hours. Cell viability analysis (**D**) and determination of drug interaction (**E**) in neuroblastoma cells treated with TNO155, lorlatinib, or TNO155 plus lorlatinib for 72 hours. **F,** Western immunoblots of Kelly and SH-SY5Y cells treated with TNO155 (1 µmol/L), lorlatinib (1 µmol/L), or combination for 24 hours. Drug concentrations are shown in Supplementary Table S1A. Synergy was calculated using the EOB model. EOB scores > 0, synergistic. *, *P* < 0.05; **, *P* < 0.01; ***, *P* < 0.001; n.s., not significant.

Innate or adaptive resistance to ALK inhibitors often leads to short-term and/or minimal responses in *ALK*-aberrant neuroblastoma tumors, thus impacting their potential utility for long-term use as single agents in patients with neuroblastoma. Because MAPK hyperactivation is a commonly observed mechanism of neuroblastoma treatment resistance (including against ALK-TKIs; refs. 11, 24, 32), we evaluated whether inactivation of MAPK signaling through SHP2 inhibition could have synergistic effects with ALK-TKIs. Cells were treated with TNO155 alone or in combination with ceritinib at similar single-agent concentrations below their respective IC_50_ (Supplementary Table S1A). Compared with single agents, the combination treatment showed increased growth inhibition in ALK^mut^, but not ALK^wt^ cell lines (Fig. 2A). Drug interactions between TNO155 and ceritinib were assessed using the EOB model (38), and EOB scores > 0 (indicating synergy for this methodology) were exclusively observed for neuroblastoma cell lines harboring *ALK* mutations (Fig. 2B). Importantly, drug combinations were effective in cells expressing the F1174L mutation (e.g., Kelly), which is typically the most resistant to the ALK inhibitors crizotinib and ceritinib. In addition, TNO155 plus crizotinib combinations exhibited similar synergistic activity in cells bearing *ALK* aberrations, including F1174L and R1275Q mutations (Supplementary Fig. S2B). To determine the mechanism(s) underlying this effect, we assessed MAPK activation status of ALK^mut^ cells treated with TNO155 plus ceritinib. While single-agent treatment decreased MAPK signaling and/or ALK activity, the combinatorial treatment showed enhanced inactivation of ALK and downstream RAS-MAPK pathway, as shown by decreased phosphorylated (p-) ALK, SHP2, and ERK1/2 (Fig. 2C). In addition, single agents showed increased apoptotic activity (cleaved-PARP) and reduced activation of PI3K/AKT/mTOR signaling (e.g., p-S6K, p-AKT), which was moderately enhanced upon combinatorial treatment (Supplementary Fig. S3A–S3C).

Because lorlatinib has broader efficacy against common neuroblastoma ALK-mutated proteins and is now included in frontline neuroblastoma trials (20, 43), we next assessed the efficacy of combining TNO155 with lorlatinib. Consistent with results using ceritinib, combination treatment with TNO155 and lorlatinib at similar drug doses exhibited cell growth inhibition and higher drug synergy as measured by EOB (scores > 4) in ALK^mut^ cells, compared with ALK^wt^ cells (Fig. 2D and E; Supplementary Fig. S3D–S3F; Supplementary Table S1A). Notably, at these examined drug doses and timepoints, the combination treatments did not lead to complete cell growth inhibition (∼30% viability). Moreover, TNO155 plus lorlatinib treatment showed marked inhibition of ALK and SHP2 activity, including inactivation of MAPK downstream signaling (Fig. 2F). In response to these treatments, we again observed decreased activation of the PI3K/AKT/mTOR pathway, and increased cell death (Supplementary Fig. S3A–S3C). In line with these results for TNO155, combinations of ALK-TKIs with another SHP2 inhibitor, SHP099, showed selective and synergistic activity in ALK^mut^ cells when treated at similar doses (Supplementary Fig. S4A–S4C; Supplementary Table S1B). Notably, SHP2 and ALK inhibitor synergy was only observed in ALK^wt^ cell lines treated with considerably higher drug doses relative to ALK^mut^ cells (3- to 62-fold higher; Supplementary Fig. S5A and S5B; Supplementary Table S1A and S1B). Taken together, these results suggest that dual inhibition of ALK and SHP2 could be an effective treatment strategy for neuroblastoma tumors harboring *ALK* aberrations.

### TNO155 Enhances ALK-TKI Antitumor Activity in Zebrafish Xenografts

**FIGURE 3:**
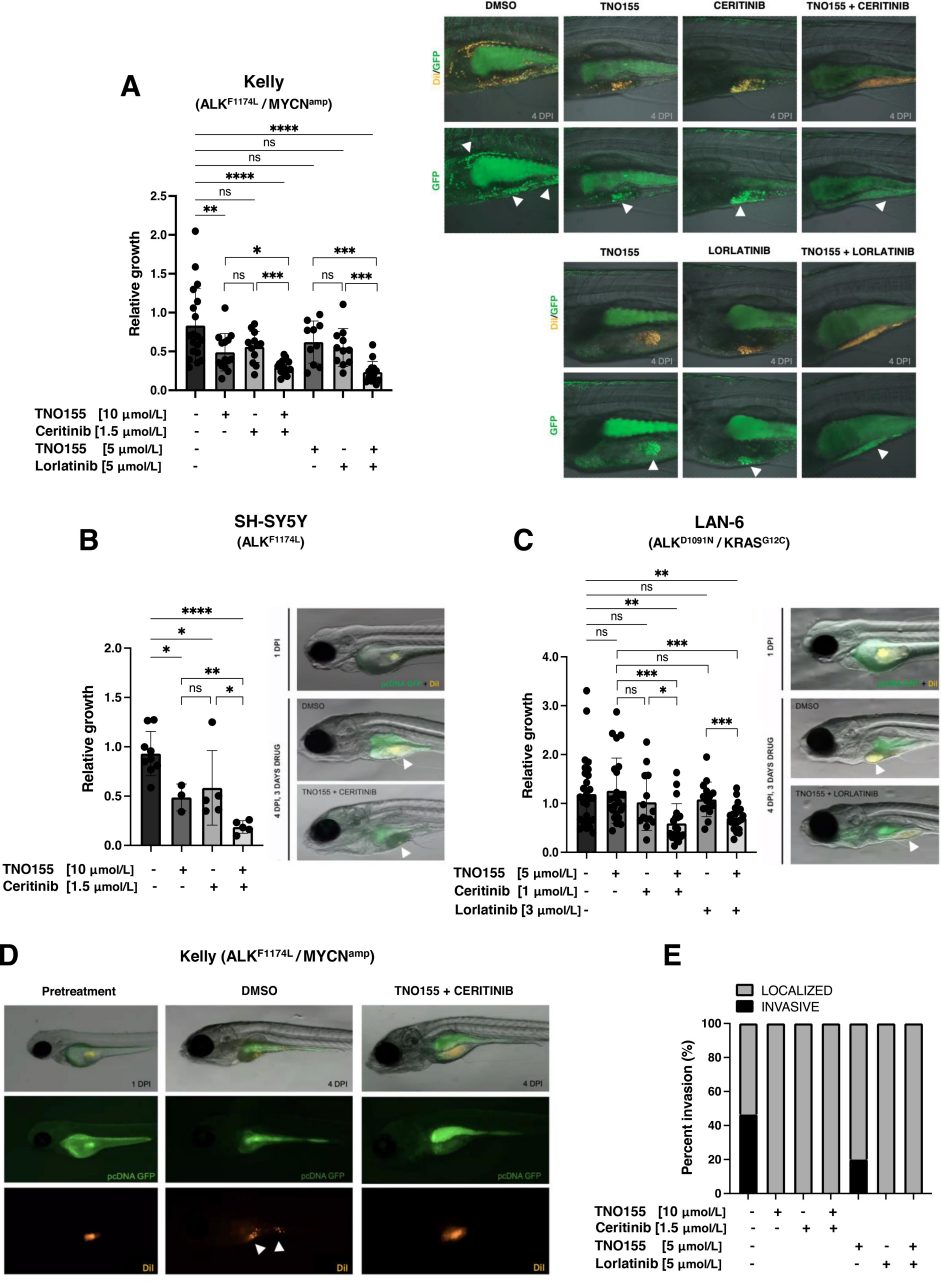
TNO155 sensitizes ALK-mutant zebrafish xenografts to ALK inhibitors. Zebrafish embryos (2 dpf) were injected with GFP-expressing Kelly (**A**), SH-SY5Y (**B**), or LAN-6 (**C**) cells costained with DiI dye. At 1 day post injection (1 DPI), xenografted larvae were submersed in TNO155, ceritinib, or lorlatinib alone or in combination treatments and incubated at 35°C for 72 hours. Tumor mass was assessed after 72 hours of drug exposure (4 DPI), and GFP fluorescence (area × pixel intensity) was quantified and normalized to pretreatment (1 DPI). Tumor cell invasion within the yolk-sac was visualized (A and **D**) and quantified (**E**) in Kelly larval xenografts treated with TNO155, ceritinib or lorlatinib alone or in combination for 72 hours. *, *P* < 0.05; **, *P* < 0.01; ***, *P* < 0.001; ****, *P* < 0.0001; ns, not significant.

To evaluate drug toxicity and efficacy *in vivo* in several differing genetic backgrounds, we administered TNO155 in combination with ceritinib or lorlatinib to larval zebrafish neuroblastoma xenograft models. We first established the MTD by submersing zebrafish embryos in drug-dissolved water containing increasing doses of TNO155, ceritinib, or lorlatinib alone or in combination (Supplementary Table S2). Kelly cells, which harbor ALK-F1174L mutation and *MYCN* amplification, were engineered to express GFP and subsequently engrafted in zebrafish embryo yolk sacs 2 dpf. Because the yolk sac is highly autofluorescent, cells were additionally stained with the long-term DiI dye to aid with tumor visualization. Imaging at 1 day post injection (DPI) showed similar tumor engraftment amongst all larvae, assessed by fluorescence intensity per area (Supplementary Fig. S6A). Engrafted larvae were treated with TNO155 alone or in combination with ceritinib or lorlatinib, and tumor growth was assessed following drug exposure. Consistent with our observations *in vitro*, combination treatments with TNO155 significantly reduced xenografted tumor growth compared with either agent alone, which only showed moderate growth inhibition *in vivo* (Fig. 3A; Supplementary Fig. S6B). These responses were also observed in an additional ALK-F1174L xenograft, SH-SY5Y (non-*MYCN* amplified) treated with TNO155 plus ceritinib (Fig. 3B). Because TNO155 has been shown to effectively reduce drug-induced feedback activation in KRAS-G12C–mutated lung and colorectal cancer cells (42), we tested combination drug treatments on LAN-6 neuroblastoma xenografts, which harbor both ALK-D1091N and KRAS-G12C mutations. Similar to previous observations in other *KRAS-G12C* cells, the addition of TNO155 significantly enhanced the antitumoral responses elicited by ceritinib or lorlatinib alone in LAN-6 xenografts at both lower and higher doses (Fig. 3C; Supplementary Fig. S6C).

We recently demonstrated that ALK has roles in neuroblastoma cell migration and metastasis (44) and thus, asked whether pharmacologic inhibition of ALK and/or SHP2 might impact cell migration. In Kelly xenografts, untreated control cells spread and migrated throughout the yolk sac and toward the tail at 4 DPI (Fig. 3A and D). Interestingly, treatments with single-agent TNO155 or combinations with ALK-TKIs led to reduced cell invasion, and localized tumor shrinkage compared with untreated controls (Fig. 3E). These effects were recapitulated *in vitro* by monitoring wound healing following treatment with SHP2 or ALK inhibitors alone or in combination (Supplementary Fig. S7A). Notably, Kelly cell migration assessed by scratch-wound closure, was reduced upon ceritinib or lorlatinib treatments alone, and further decreased when combined with the SHP2 inhibitor (Supplementary Fig. S7B). These results suggest that concurrent SHP2/ALK inhibition in ALK-mutant neuroblastomas can reduce tumor growth *in vivo* and potentially impair cell migration.

### TNO155 and Lorlatinib Combination Delays Tumor Growth in Murine Xenografts

**FIGURE 4:**
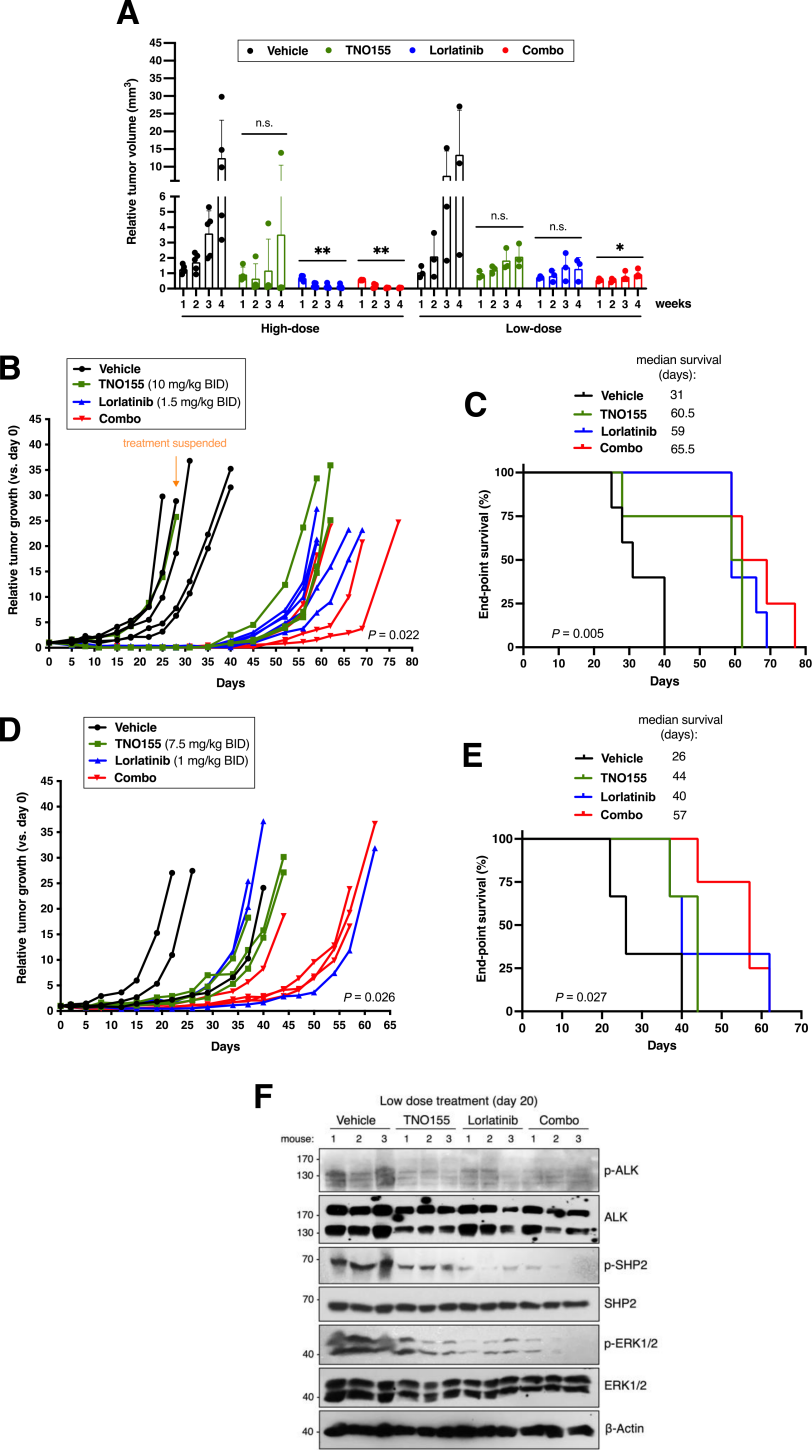
TNO155 and lorlatinib treatment delays tumor growth in ALK-mutant mouse xenografts. **A,** Tumor volumes of Kelly xenografts treated orally with high- or low-dose regimens of TNO155, lorlatinib, combination treatment (Combo), or vehicle control for 4 weeks. Tumor growth (**B**) and Kaplan–Meier survival curves (**C**) of Kelly xenografts treated with high-dose vehicle (*n* = 5), TNO155 (*n* = 4), lorlatinib (*n* = 5), or combination (Combo, *n* = 4) treatments. Tumor growth (**D**) and Kaplan–Meier survival curves (**E**) of Kelly xenografts treated with low-dose vehicle (*n* = 3), TNO155 (*n* = 3), lorlatinib (*n* = 3), or combination (Combo, *n* = 4) treatments **F,** Protein expression analyses of tumor lysates treated with vehicle control (*n* = 3), TNO155 (7.5 mg/kg twice per day, *n* = 3), lorlatinib (1 mg/kg twice per day, *n* = 3), or combination (Combo, *n* = 3) for 20 days. *, *P* < 0.05; **, *P* < 0.01; n.s., not significant.

To evaluate the tolerability of combined TNO155 and lorlatinib treatment in murine neuroblastoma models, we first assessed MTDs of each agent alone and in combination. At the MTD determined for TNO155 (20 mg/kg twice per day) and lorlatinib (5 mg/kg twice per day), we observed no drug-related toxicities with single agents or combination regimens for at least 21 days. To assess drug efficacy *in vivo*, tumor growth was monitored in mice bearing subcutaneous ALK-F1174L Kelly xenografts and treatment was assessed using two different dosing protocols, with high- or low-dose regimens of TNO155, lorlatinib, or combination (Table 2). At high doses, TNO155 treatment led to variable moderate responses, whereas lorlatinib or the combination treatment both resulted in significant tumor growth inhibition after 4 weeks of treatment (Fig. 4A). Comparatively, low-dose regimen treatments with TNO155 or lorlatinib only partially reduced tumor burden compared with vehicle control, but importantly, significant growth inhibition was observed with the combination (Fig. 4A).

**TABLE 2:** Treatment regimens administered to Kelly xenografts

Treatment	High-dose	Low-dose
TNO155	10 mg/kg BID	7.5 mg/kg BID
Lorlatinib	1.5 mg/kg BID	1 mg/kg BID
Combo	10 + 1.5 mg/kg BID	7.5 + 1 mg/kg BID
**Regimen**		
Schedule	5 days/week	5 days/week
Duration	4 weeks	9 weeks

Abbreviation: BID, twice daily.

For the high-dose regimen, treatment was suspended after 4 weeks once regression was observed, and tumor regrowth was monitored for up to 50 days to examine drug activity and durability. Interestingly, tumor growth remained negligible for at least 10 days following treatment discontinuation. While single agents effectively delayed tumor burden, growth inhibition was slightly more evident in tumors treated with TNO155 plus lorlatinib combination (Fig. 4B; Supplementary Fig. S8A). Delayed tumor growth correlated with increased survival of mice treated with single agents, and to a larger extent, with combination treatment (Fig. 4C). No adverse side effects, including significant weight changes were detected (Supplementary Fig. S8B).

The low-dose treatment regimen of TNO155, lorlatinib, or the combination did not lead to complete tumor regression, and thus, treatment was administered until endpoint (9 weeks). Lower doses of single-agent TNO155 or lorlatinib alone still led to modest tumor growth inhibition (albeit less effectively than higher doses) compared with control; however, combination treatment significantly delayed tumor growth (Fig. 4D; Supplementary Fig. S8C and S8D). Survival of mice treated with combination therapy was increased compared with control and single agents (Fig. 4E). Notably, the median survival between single and combination treatments was extended by 13 to 17 days at low-dose regimens compared with 5 to 6.5 days at high-dose treatments.

To investigate the pharmacodynamics of TNO155 and lorlatinib alone or in combination we harvested murine tumors treated for 20 days with low-dose combination regimen. Similar to our observations *in vitro*, single agents diminished ALK and SHP2 activity, which lead to decreased MAPK and mTOR signaling via ERK and S6K inactivation, respectively (Fig. 4F; Supplementary Fig. S8E). Furthermore, lysates from tumors treated with TNO155 plus lorlatinib showed potent reduction of MAPK signaling, with negligible p-ERK1/2 levels compared with other treatment groups. Interestingly, tumors treated with the drug combination for over 30 days still showed MAPK pathway inactivation, despite developing insensitivity to SHP2 inhibition alone (Supplementary Fig. S8F). Taken together, these results suggest that high-dose regimens of TNO155 or lorlatinib alone might be sufficient to delay tumor growth of *ALK* mutated tumors; however, low-dose combination treatments could impact tumor responses more significantly due to their ability to treat for longer periods with fewer toxicities.

### Dual SHP2/ALK Inhibition Restores Sensitivity in Lorlatinib-resistant Neuroblastoma

**FIGURE 5:**
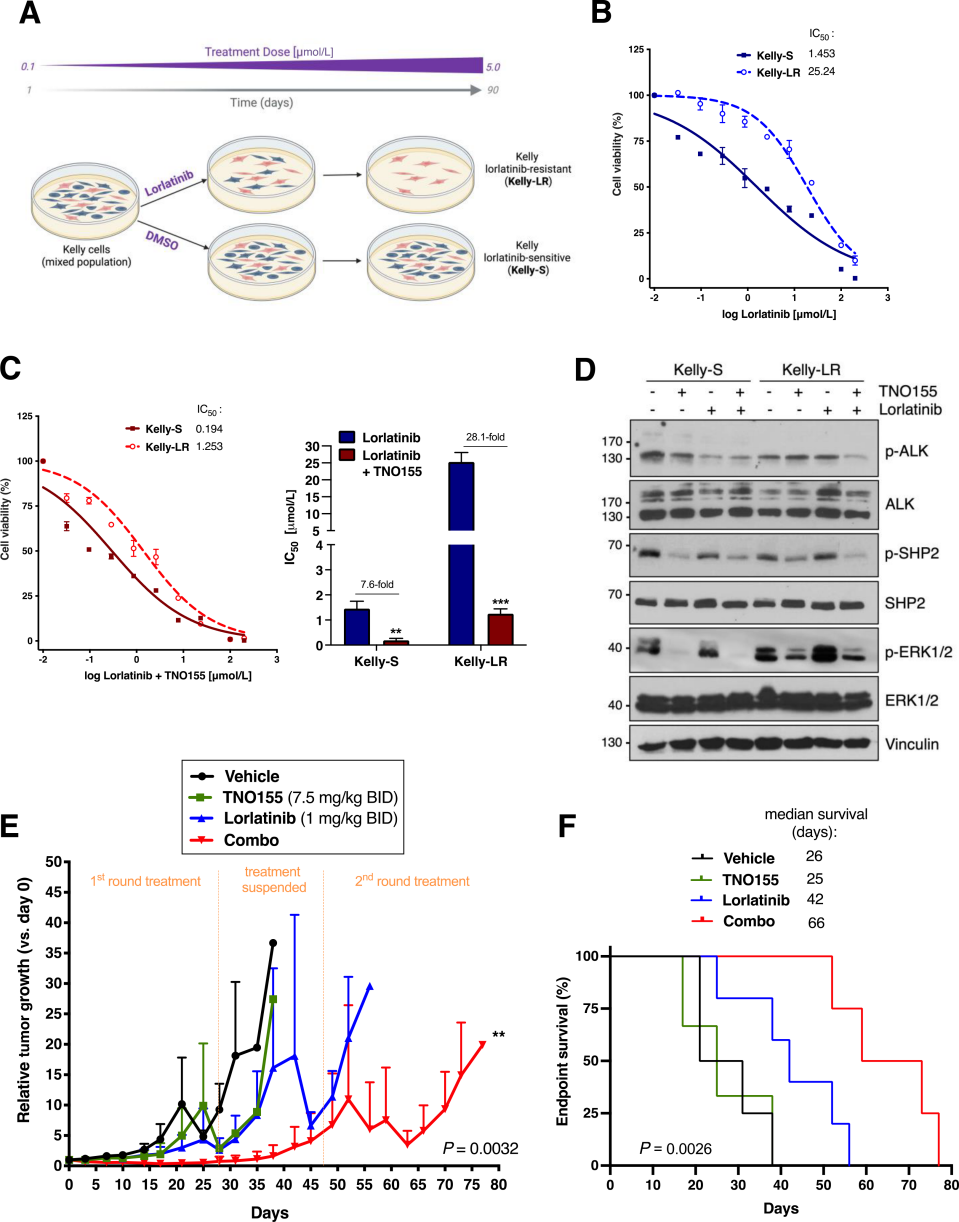
Dual SHP2/ALK inhibition resensitizes lorlatinib-resistant ALK^F1174L^ cells and reduces tumor regrowth. **A,** Kelly cells were treated with increasing doses of lorlatinib (0.1–5.0 µmol/L) or DMSO over 90 days to select for subpopulations of lorlatinib-resistant (Kelly-LR) or -sensitive (Kelly-S) cells. Cell lines were maintained under chronic exposure to lorlatinib or DMSO. Created with BioRender.com. Cell viability (alamarBlue) and IC_50_ was assessed in Kelly-S and Kelly-LR cells treated with increasing concentrations of lorlatinib alone (**B**) or lorlatinib plus TNO155 (**C**) for 72 hours. A maximum of 150 µmol/L of either or both drugs was serially diluted (1:3) to assess wide-range dose efficacy (0–150 µmol/L, doses of 0.0076, 0.0228, 0.0685, 0.205, 0.617, 1.851, 5.555, 16.666, 50, and 150 µmol/L). **D,** Western immunoblots of Kelly-S and Kelly-LR cells treated with TNO155 (1.5 µmol/L), lorlatinib (1 µmol/L), or combination treatment for 6 hours. Tumor growth (**E**) and Kaplan–Meier survival curves (**F**) of Kelly xenografts treated with two rounds of low-dose vehicle control (*n* = 4), TNO155 (*n* = 3), lorlatinib (*n* = 5), or combination treatment (Combo, *n* = 4) for 4 weeks each, with a 3 week drug break in between each round. **, *P* < 0.01; ***, *P* < 0.001.

Although lorlatinib treatment has reportedly been more successful at delaying ALK inhibitor resistance compared with other ALK-TKIs, certain cancer models including relapsed ALK-F1174L neuroblastomas have been shown to eventually acquire resistance after long-term treatment (22–24, 45, 46). To examine whether TNO155 could restore sensitivity in resistant ALK-F1174L neuroblastomas, we first generated a lorlatinib-resistant cell line model based on previously reported methodologies (24, 47, 48). Kelly cells were exposed to gradually increasing doses (0.1–5 µmol/L) of lorlatinib or vehicle (DMSO) over 90 days, and subpopulations of lorlatinib-resistant (Kelly-LR) or lorlatinib-sensitive (Kelly-S) cells were selected (Fig. 5A). Acquired lorlatinib resistance was confirmed after observing an approximately 17-fold IC_50_ increase in Kelly-LR cells, but negligible changes in Kelly-S cells as compared with parental Kelly cells (Fig. 5B; Table 1). Interestingly, the addition of TNO155 restored basal sensitivity to lorlatinib in Kelly-LR cells, as the IC_50_ of the combined treatment (1.25 µmol/L) was lower than that of Kelly-S cells treated with lorlatinib or TNO155 alone (1.45 µmol/L or 2.66 µmol/L, respectively; Fig. 5C; Supplementary Fig. S9A). We next evaluated treatment-dependent ALK signaling differences between sensitive and resistant cells. Consistent with our cell viability studies, lorlatinib treatment no longer impacted ALK activation (p-ALK) in Kelly-LR cells compared with Kelly-S; however, TNO155 plus lorlatinib combination markedly reduced p-ALK to levels similar to those observed in Kelly-S cells, and resulted in decreased MAPK, mTOR, and PI3K downstream signaling (Fig. 5D; Supplementary Fig. S9B). Interestingly, we observed higher basal expression of p-ERK1/2 in the Kelly-LR cell subpopulation, compared with Kelly-S cells. In contrast, neither p-S6K nor p-AKT showed enhanced expression in Kelly-LR cells, thus suggesting that lorlatinib resistance might be more specifically associated with aberrant RAS-MAPK signaling. Taken together, these results show that lorlatinib-resistant cells can be resensitized upon combination with TNO155.

To determine whether TNO155 could restore sensitivity to lorlatinib *in vivo*, we treated Kelly xenografts with TNO155 (7.5 mg/kg twice per day), lorlatinib (1 mg/kg twice per day), or combination treatments for 4 weeks (first round), followed by a 3-week treatment suspension, and subsequent 4-week retreatment (second round). During the first round of treatment, we observed moderate growth inhibition in tumors treated with TNO155 or lorlatinib, whereas combination treatment showed a complete tumor regression response (Fig. 5E; Supplementary Fig. S9C and S9D). Upon suspension of treatment, both TNO155 and lorlatinib cohorts showed more accelerated tumor regrowth rates compared with the combination group. Consistent with our *in vitro* observations, tumors previously treated with lorlatinib showed increased tolerance to the second round lorlatinib treatment, whereas most combination-treated tumors remained moderately responsive to treatment and showed increased overall survival (Fig. 5F). All remaining tumors reached endpoint shortly after the second round of treatment was suspended. Notably, all mice treated with TNO155 reached endpoint before treatment was restarted.

### Aberrant RAS-MAPK Signaling Associates with Lorlatinib Resistance

**FIGURE 6:**
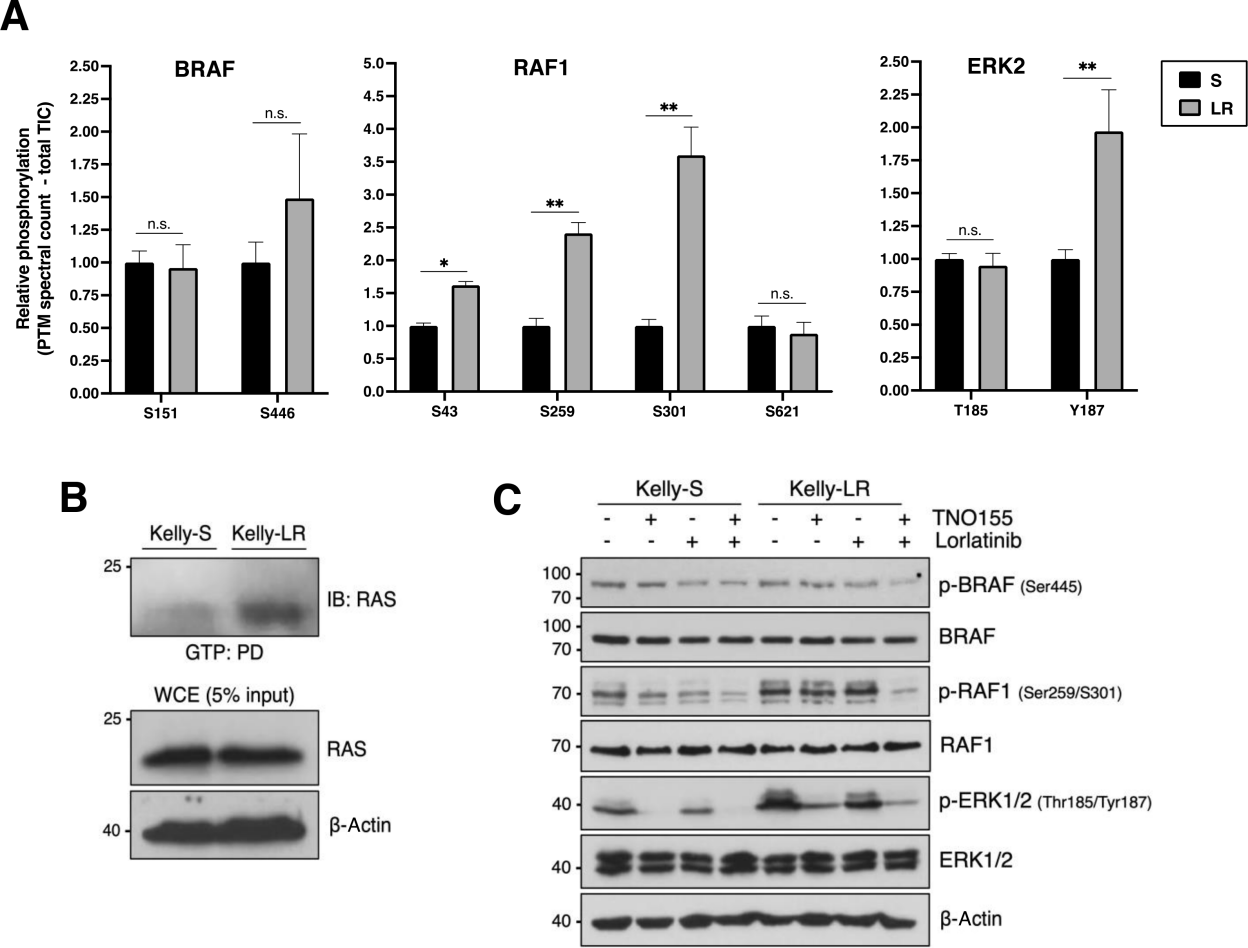
Alterations in RAS-MAPK pathway associate with lorlatinib resistance. **A,** Abundance of specific BRAF, RAF1, and ERK2 phospho-sites identified by LC/MS-MS in Kelly-LR samples relative to Kelly-S. **B,** GTP pulldown (PD) of WCE lysates of Kelly-S and Kelly-LR cells. IB, immunoblot. **C,** Western immunoblots of Kelly-S and Kelly-LR cells treated with TNO155 (1.5 µmol/L), lorlatinib (1 µmol/L) or combination treatment for 6 hours. *, *P* < 0.05; **, *P* < 0.01; n.s., not significant.

To further explore the mechanisms underlying this acquired lorlatinib resistance, we performed WGS analysis of the Kelly-S and Kelly-LR subpopulations and identified 26 genes exclusively altered in the Kelly-LR subpopulation (Supplementary Table S3). Among these, aberrations in *BRAF*, *MEGF6 (EGFL6)*, *DAXX*, *RAG1, DDX54,* and *ASPSCR1* genes have been previously linked to neuroblastoma pathogenicity, drug resistance and/or RAS-MAPK activation (refs. 22, 49–55; Table 3). Interesting, BRAF-G466E mutation encodes a kinase impaired protein that has previously shown to bind more tightly to activated RAS and promote hyperactivation of MAPK components (56). To assess the phosphoproteomics landscape of these cell subpopulations, we also performed LC/MS-MS analyses of Kelly-S and Kelly-LR peptides that were enriched for pSTY sites. Among the upregulated peptides identified in Kelly-LR cells, we observed increased activation of several RAS-MAPK downstream effectors, including BRAF (p-S446), RAF1 (p-S259/S301), and ERK2 (p-Y187; Fig. 6A). In addition, we observed higher levels of RAS-GTP–bound proteins in Kelly-LR cells compared with Kelly-S, thus suggesting that RAS activity is increased in *BRAF*-aberrant Kelly-LR cells (Fig. 6B). Finally, as further validation and consistent with our phosphoproteomics data, Western immunoblots showed that p-RAF1 (S259/S301) and p-ERK2 (Y187) were more highly expressed in Kelly-LR cells (Fig. 6C). Interestingly, MAPK hyperactivation in Kelly-LR cells impacted the ability of lorlatinib to reduce p-RAF1 and p-ERK levels compared with Kelly-S cells; however, resensitization and overall MAPK inactivation was observed upon addition of TNO155. These results suggest that *BRAF* mutation is, at least in part, mediating MAPK hyperactivation in Kelly-LR cells, which likely contributes to reduced lorlatinib efficacy, but can subsequently be resensitized with dual ALK/SHP2 inhibition.

**TABLE 3:** Detectable DNA mutations and variant allele frequencies (VAF) in selected genes exclusively identified in the Kelly-LR cell subpopulation

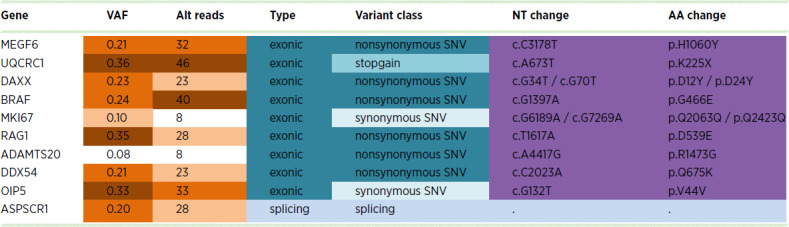						

Abbreviations: NT, nucleotide; AA, amino acid; SNV, single nucleotide variant.

NOTE: Genes listed according to chromosomal location (see complete list in Supplementary Table S3).

## Discussion

Neuroblastoma recurrence often presents as metastatic and/or therapy-resistant tumors and remains a major clinical challenge. The identification of *ALK* mutations in subsets of neuroblastoma primary tumors and more commonly in relapsed samples (5, 6, 9–11) resulted in trials of ALK inhibitors and development of newer compounds with better efficacy against activating missense mutations. However, pediatric clinical trials have shown limited long-term single-agent ALK-TKI activity for neuroblastoma, because innate or acquired resistance often leads to drug inefficacy and tumor recurrence (14, 17, 20, 22, 57). Mechanisms of ALK-TKI resistance include acquired mutations and hyperactivation of MAPK or PI3K signaling, both of which have been shown to be activated, in part, by SHP2 and dampened upon its pharmacologic targeting (23–25, 58). Furthermore, we have previously shown that combinatorial inhibition of SHP2 with other MAPK or PI3K components results in synergistic growth inhibition of neuroblastoma and other pediatric malignancies bearing *RAS-* or *PI3K*-activating mutations (30, 59). In this study, our results provide evidence for the use of SHP2 in combination with ALK-TKIs in high-risk and/or relapsed neuroblastoma tumors harboring *ALK* mutations. In addition, we provide extensive *in vitro* studies and the first *in vivo* data in neuroblastoma models evaluating TNO155, which is currently being studied in early phase clinical trials in adults with lung or colorectal cancer, melanoma, esophageal or head/neck squamous cell carcinoma, and other resistant solid tumors (NCT03114319, NCT04292119, NCT04699188). Some of these trials include combinations with ALK-TKIs for patients with evidence of *ALK* translocations.

In recent years, cross-talk mechanisms between SHP2 and ALK have become more evident. In *ALK*-addicted neuroblastoma cells, integrated proximal proteomics studies identified SHP2 as a direct interactor of ALK phospho-tyrosine residues, with further validation revealing that SHP2 activation and subsequent protein–protein interaction is abrogated upon ALK inhibition following ALK-TKI treatment (27, 60). This functional dependency on ALK activation provides rationale for targeting SHP2 in tumors with aberrant ALK signaling. Here, we show that SHP2 inhibitors SHP099 and TNO155 show higher selectivity in ALK-mutant neuroblastoma cells compared with those with wild-type status. Importantly, TNO155 showed substantially higher single-agent antitumor activity than SHP099, and much lower doses were required to achieve similar effects in neuroblastoma cells and tumors when compared with our published data for SHP099 (30). This enhanced efficacy of TNO155 compared with SHP099 was also recently reported in malignant peripheral nerve sheath tumors (61). The selective differences in TNO155 sensitivity that we observed between ALK mutated and nonmutated neuroblastoma cells resemble those reported for ALK-TKI treatments (13); however, our results reveal no substantial differences based on specific *ALK* missense mutations. Furthermore, dual targeting of SHP2 (e.g., TNO155, SHP099) and ALK (e.g., ceritinib, lorlatinib) resulted in synergistic reduction of ALK-mutant cell viability and inhibition of MAPK and PI3K/AKT/mTOR downstream signaling. These results are in line with reports identifying SHP2 as a *bona fide* mediator of survival in ALK-mutant neuroblastoma cells and *in vitro* synergy between SHP099 and lorlatinib (36). Moreover, SHP2 inhibition has recently been shown to reduce MAPK and mTOR signaling via inactivation of ERK, mTORC1, and S6K in neuroblastoma cells and tumors with low NF1 expression (41).

The clinical potential of concurrent SHP2/ALK inhibition remains largely unexplored. While a few *in vivo* studies evaluating SHP099 alone or combined SHP2/ALK inhibition have shown promising responses in a subset of neuroblastomas with *NF1* loss (including several harboring *ALK* mutations), or in *ALK*- or *ROS1*-rearranged lymphomas and lung tumors, respectively (33, 34, 39, 41), thus far no preclinical drug combination evaluations have been conducted in *ALK*-missense mutant tumors, including neuroblastoma. We also explored zebrafish larval xenograft models as they enable time and cost-effective, small scale (low cell counts), and medium-to-high throughput chemical screenings *in vivo* (62). Our drug studies showed that both TNO155 combinations with ceritinib or lorlatinib effectively reduced tumors in three different larval xenograft models. Notably, Kelly xenografts and *in vitro* cell models harboring an *ALK* mutation and *MYCN* amplification showed increased cell migration at baseline, which was reduced or abrogated after single or combinational SHP2/ALK inhibition treatment. To date, this is the first evidence demonstrating changes in neuroblastoma invasion and potentially metastasis in response to treatment with drugs targeting both ALK and SHP2. These results are in line with previous reports showing that *ALK* and *MYCN* cooperate in transgenic neuroblastoma zebrafish pathogenesis (63), and that ALK knockdown suppresses MYCN-induced cell migration (64). We further show that long-term TNO155 and lorlatinib treatment is tolerable in mice, reduces murine tumor growth, and increases survival, thus complementing our short-term observations in zebrafish and providing evidence of the effective transability between these *in vivo* models.

Acquired resistance to long-term single-agent ALK-TKI treatment is a major challenge in patients who initially experience clinical responses. Previous reports support that mechanisms of resistance are often associated with acquired mutations that lead to MAPK reactivation. Thus, SHP2 represents an optimal therapeutic target to prevent, delay, or reverse ALK-TKI resistance. Moreover, SHP2 mediates the activation of several receptor tyrosine kinases and downstream effectors of multiple signaling pathways (e.g., ALK-MAPK, PI3K/AKT/mTOR), and has been identified as a common resistance node in subsets of *ALK*-rearranged lung cancers (23, 25, 33, 42). Here, using WGS and phosphoproteomic approaches we have identified altered genes (e.g., *BRAF* and *MEGF6*) that are predicted to upregulate the RAS-MAPK pathway, as well as several MAPK downstream effectors that are differentially activated in the lorlatinib-resistant, Kelly-LR, cell subpopulation model that we generated. These findings are in line with other reports that have shown the emergence of RAS-MAPK aberrations as a mechanism of acquired resistance to lorlatinib in cell models and patients (22–25). Consistent with other reports that have shown reversal of ALK-TKI resistance in *ALK*-translocated lymphoma and lung cancers (33, 34), our studies provide evidence that TNO155 can restore ALK-TKI sensitivity in neuroblastoma cells and tumors harboring *ALK* missense mutations that have acquired resistance to lorlatinib overtime. Although our combination treatments were not able to achieve complete tumor regression, we observed decreased tumor burden and increased survival. These results warrant further exploration and validation in additional preclinical models to potentially support the development of clinical trials for patients with relapsed neuroblastoma, especially given recent evidence demonstrating the emergence of resistance mutations detected in ctDNA of patients treated with ALK-TKIs (21, 22).

Overall, our studies provide *in vitro* and *in vivo* support for single-agent TNO155 treatment in neuroblastoma and demonstrate the efficacy and tolerability of TNO155 combinations with ALK-TKIs in several *ALK*-mutant neuroblastoma models. Dual SHP2/ALK inhibition was effective in both cell lines and tumors that are sensitive to ALK-TKIs and those that are more tolerant, and somewhat resistant, to ALK-TKIs; and importantly, larval zebrafish xenografts accurately predicted efficacy in murine models. In previous reports, we had shown that combined SHP2 inhibition restores sensitivity to RAS-MAPK or PI3K inhibitors in neuroblastoma and other pediatric malignancies harboring *NRAS* or *PI3K* mutations (30, 59), thus informing further development of potentially useful treatment regimens for patients with relapsed neuroblastoma based on the genetic profile of their tumors. Here, we also show that SHP2 inhibitors can be effectively combined with ALK inhibitors in targeting *ALK*-mutated neuroblastoma, including those with acquired resistance to ALK-TKI therapies. Interestingly, it has been recently reported that SHP2 inhibition can sensitize oncogene-addicted tumors to retreatment with targeted therapies, including ALK inhibitors, for *ALK* fusion–positive lung cancer; these effects were observed in preclinical models and patients (65). In summary, our results suggest that SHP2 combination regimens are a promising therapeutic strategy to further investigate as a possible regimen for patients with neuroblastoma and other solid tumors with multiple genetic aberrations in the RAS/MAPK/SHP2 pathways, to provide opportunities to enhance drug sensitivity and potentially impact resistance to single agents. These findings provide evidence to support additional evaluations of lorlatinib combinations with the SHP2 inhibitor TNO155, which is currently in phase I/II trials for adult cancers, for the treatment of relapsed *ALK*-mutant neuroblastoma, including those with acquired resistance to ALK inhibitors.

## Supplementary Material

Supplementary Materials and Methods Includes additional information regarding cell culture, scratch wound-healing assays, antibodies, and mass spectrometry and phosphoproteomics analyses.

Table S1 Table S1. Drug doses for ALK-TKI combinations with TNO155 or SHP099 for synergy assessment.

Table S2 Table S2. Maximal tolerated doses (MTD) for TNO155 alone and in combination with ceritinib or lorlatinib in zebrafish embryos.

Table S3 Table S3. Genetic aberrations detected in Kelly-LR cell subpopulation.

Figure S1 Figure S1. ALK aberrant neuroblastoma cell lines are sensitive to SHP099.

Figure S2 Figure S2. ALK-TKIs synergize with TNO155 in ALK mutant neuroblastoma cells.

Figure S3 Figure S3. TNO155 and ALK-TKI treatments increase apoptosis, reduce PI3K/AKT/mTOR signaling, and decrease growth of ALK mutant cells.

Figure S4 Figure S4. SHP099 synergizes with ALK-TKIs in ALK mutant neuroblastoma cells.

Figure S5 Figure S5. High doses of SHP2 inhibitors sensitize ALK wildtype neuroblastoma cells to ALK-TKIs.

Figure S6 Figure S6. Assessment of TNO155 and ALK-TKI efficacy in zebrafish embryos.

Figure S7 Figure S7. Dual SHP2 and ALK inhibition reduces neuroblastoma cell migration.

Figure S8 Figure S8. TNO155 plus Lorlatinib treatment decreases tumor burden in ALKF1174L murine xenografts.

Figure S9 Figure S9. TNO155 re-sensitizes lorlatinib-resistant ALKF1174L cells and tumors.
