# Supplementary material for: SHP2 Inhibition with TNO155 Increases Efficacy and Overcomes Resistance of ALK Inhibitors in Neuroblastoma
Source: Cancer Res Commun. 2023 Dec 27;3(12):2608–22. doi: 10.1158/2767-9764.CRC-23-0234 (PMC10752212; doi:10.1158/2767-9764.CRC-23-0234)
Supplement: Supplementary Materials and Methods — Includes additional information regarding cell culture, scratch wound-healing assays, antibodies, and mass spectrometry and phosphoproteomics analyses. [file crc-23-0234-s01.pdf]

SHP2 INHIBITION WITH TNO155 INCREASES EFFICACY AND OVERCOMES RESISTANCE OF ALK INHIBITORS IN NEUROBLASTOMA

Ivette Valencia-Sama, Lynn Kee, Gabriella Christopher, Michael Ohh, Mehdi Layeghifard, Adam Shlien, Madeline N. Hayes and Meredith S. Irwin

SUPPLEMENTARY MATERIALS AND METHODS

Cell culture

IMR-32 and CHP-212 cells were cultured in EMEM (Wisent), and SK-N-AS and NGP cells in DMEM (Wisent), with both media supplemented with 10% FBS (Wisent), 0.1mM nonessential amino acids (Gibco), and 1mM sodium pyruvate (Gibco). COG-N-415 and COG-N-519 cells were cultured in IMDM (Wisent) supplemented with 20% FBS (Wisent), 3mM L-glutamine (Gibco), and 1x ITS (5 µg/mL insulin, 5 µg/mL transferrin, 5 ng/mL selenous acid). Other cell lines were cultured in RPMI medium (Wisent) supplemented with 10% FBS, 3mM L-glutamine (Gibco), and 1mM sodium pyruvate (Gibco). Cells were grown at 37ºC in 5% CO2 tissue culture incubators.

Scratch wound-healing assays

Kelly cells were grown to confluency in full-serum media and scratch-wound induced with pipette tips. Cells were treated with DMSO (control), SHP099, ceritinib, lorlatinib or combination treatments, and images were captured (0 and 72 hours). Distance migrated was measured using ImageJ software, normalized to pre-treatment, and expressed as percent wound closure.

Antibodies

Anti-mouse antibodies: Vinculin (Millipore #05386, 1:2000), β-actin (Sigma #A5316, 1:2000), ERK1/2 (Cell Signaling Technologies #9107, 1:1000), p53 (Millipore #OP45, 1:1000), pan-RAS (Millipore #OP40, 1:500), and MYCN (Millipore #OP13L, 1:500).

Anti-rabbit antibodies: p-SHP2 (Y542) (#3751, 1:1000), SHP2 (#3752, 1:1000), p-ERK1/2 (T202/Y204) (#9101,1:1000), p-ALK (Y1604) (#3341, 1:500), ALK (#3333, 1:1000), cleaved PARP (#9541, 1:1000), PARP (#9532, 1:1000), p-BRAF (S445) (#2696, 1:1000), BRAF (#9433, 1:1000), p-CRAF (S259) (#9421, 1:1000), p-CRAF (S289/296/301) (#9431, 1:1000), CRAF (#9422, 1:1000), p-p70 S6K (Thr389) (#9234, 1:1000), p70 S6K (#9202, 1:500), p-p90AKT (#9272, 1:1000), and p-AKT (T308) (#13038, 1:1000) were obtained from Cell Signaling Technologies. p-SHP2 (Y542) (#ab62322, 1:1000) and p-ALK (Y1507) (#ab73996, 1:1000) were obtained from Abcam. p-ERK1/2 (T185/Y187) (#700012, 1:2000) was obtained from Invitrogen.

Mass spectrometry and phosphoproteomics analyses

Samples were reduced with DTT (10 mM, 60°C, 1 hour), alkylated with iodoacetamide (20 mM, room temperature, 45 min, dark), and digested overnight at 37°C with trypsin (Cell Signaling PTM Scan Trypsin, #56296, 20 µg per sample, 1:15). Protein precipitation and pSTY enrichment was performed with Pierce High-Select Fe-NA Phosphopeptide Enrichment Kit (Thermo # A32992), and samples were diluted in ice cold acetone (1:10, -20°C, 1.5 hours) and resuspended in 2% ACN, 0.1% formic acid. Peptides were dried by vacuum centrifugation, desalted on C18 ziptips (Millipore) using a DigestPro MSi (Intavis Bioanalytical Instruments), and dried again by vacuum centrifugation before resuspension in Buffer A (0.1% formic acid v/v in water). Samples were analyzed by liquid chromatography tandem mass spectrometry (LC-MS/MS) using an Evosep One LC system 15 SPD Method (3 µg peptide on tip) and an Orbitrap Fusion™ Lumos™ Tribrid™ Mass Spectrometer (Thermo Fisher Scientific). The LC portion of the analysis consisted of a 18 min linear gradient running 3-20% of Buffer A to Buffer B (0.1% formic acid v/v in acetonitrile), followed by a 31 min linear gradient running 20-35% of Buffer A to Buffer B, a 2 min ramp to 100% Buffer B and 9 min hold at 100% Buffer B, all at a flow rate of 250 nL/min. Samples were loaded into a 75 µm x 2 cm Acclaim PepMap 100 Pre-column followed by a 150 µm ID x 15 cm PepMax RSLC EASY-Spray analytical column filled with 1.9 µM C18 beads (Thermo Fisher Scientific). MS1 acquisition resolution was set to 120000 with an automatic gain control (AGC) target value of 4 x 105 and maximum ion

injection time (IT) of 50 ms for a scan range of m/z 375-1500, with dynamic exclusion set to 10 s. Isolation for MS2 scans was performed in the quadrupole with an isolation window of m/z 0.7. MS2 scans were performed in the ion trap with maximum ion IT of 10 ms, AGC target value of 1 x 104, and higher-energy collisional dissociation (HCD) activation with an NCE of 30.

MS raw files were analyzed using PEAKS Studio software (Bioinformatics Solutions Inc.) and Proteome Discoverer (version 2.5.0.400) and fragment lists searched against the human UniProt Reference database (Uniprot_UP000005640_Human_15092020.fasta, downloaded Aug. 15, 2023). For both search algorithms, the parent and fragment mass tolerances were set to 50 ppm and 0.02 Da, respectively, and only complete tryptic peptides with a maximum of 3 missed cleavages were accepted. Carbamidomethylation of cysteine was specified as a fixed modification; deamidation of asparagine and glutamine, oxidation of methionine, acetylation of the protein N-terminus, and phosphorylation of serine, threonine, and tyrosine residues were specified as variable modifications.
