# Supplementary material for: SHP2 Inhibition with TNO155 Increases Efficacy and Overcomes Resistance of ALK Inhibitors in Neuroblastoma
Source: Cancer Res Commun. 2023 Dec 27;3(12):2608–22. doi: 10.1158/2767-9764.CRC-23-0234 (PMC10752212; doi:10.1158/2767-9764.CRC-23-0234)
Supplement: Figure S1 — ALK aberrant neuroblastoma cell lines are sensitive to SHP099. [file crc-23-0234-s05.pdf]

Figure S1

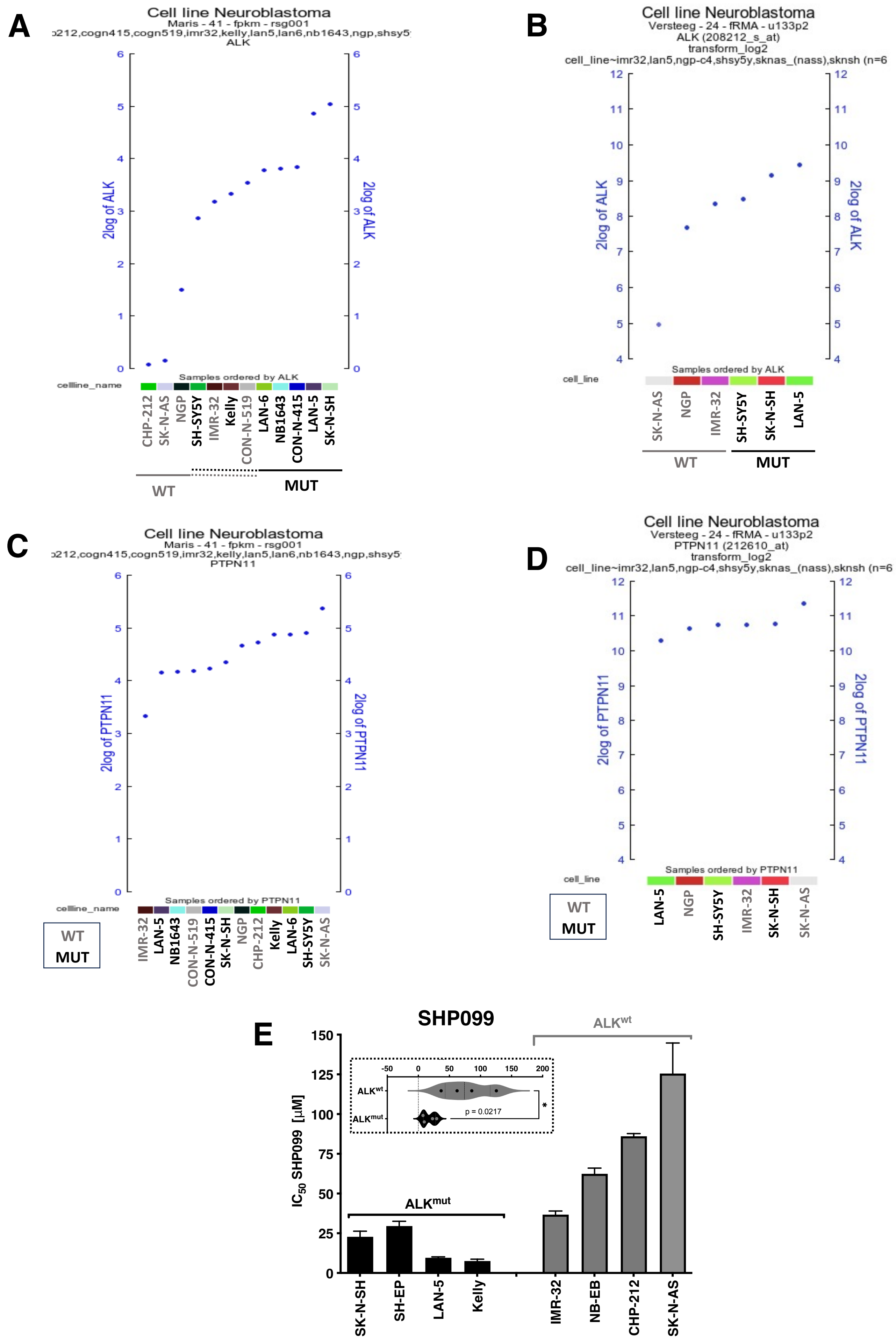

A
Cell line Neuroblastoma
Maris - 41 - fpkm - rsg001
ALK
2log of ALK
Samples ordered by ALK
cellline_name
CHP-212
SK-N-AS
NGP
SH-SY5Y
IMR-32
Kelly
CON-N-519
LAN-6
NB1643
CON-N-415
LAN-5
SK-N-SH
WT
MUT
B
Cell line Neuroblastoma
Versteeg - 24 - fRMA - u133p2
ALK (208212_s_at)
transform_log2
cell_line~imr32,lan5,ngp-c4,shsy5y,sknas_(nass),sknsh (n=6
2log of ALK
Samples ordered by ALK
cell_line
SK-N-AS
NGP
IMR-32
SH-SY5Y
SK-N-SH
LAN-5
WT
MUT
C
Cell line Neuroblastoma
Maris - 41 - fpkm - rsg001
PTPN11
2log of PTPN11
Samples ordered by PTPN11
cellline_name
WT
MUT
IMR-32
LAN-5
NB1643
CON-N-519
CON-N-415
SK-N-SH
NGP
CHP-212
Kelly
LAN-6
SH-SY5Y
SK-N-AS
D
Cell line Neuroblastoma
Versteeg - 24 - fRMA - u133p2
PTPN11 (212610_at)
transform_log2
cell_line~imr32,lan5,ngp-c4,shsy5y,sknas_(nass),sknsh (n=6
2log of PTPN11
Samples ordered by PTPN11
cell_line
WT
MUT
LAN-5
NGP
SH-SY5Y
IMR-32
SK-N-SH
SK-N-AS
E
SHP099
ALKwt
ALKmut
p = 0.0217
*
IC50 SHP099 [µM]
SK-N-SH
SH-EP
LAN-5
Kelly
IMR-32
NB-EB
CHP-212
SK-N-AS

Figure S1. *ALK* aberrant neuroblastoma cell lines are sensitive to SHP099.

A-D, Gene expression analysis of *ALK* and *PTPN11* (SHP2) in a panel of neuroblastoma cell lines with *ALK* wildtype (WT) or mutant (MUT) status. Graphs were generated using the R2: Genomics Analysis and Visualization Platform (http://r2.amc.nl) "Cell line Neuroblastoma - Maris - 41 - FPKM – rsg001" (GEO: GSE89413) (A and C), and "Cell line Neuroblastoma - Versteeg - 21 - fRMA – u133p2" (GEO: GSE28019) (B and D) datasets. **E**, Calculation of IC_{50} in neuroblastoma cells with *ALK* wildtype (ALK^{wt}) or mutant (ALK^{mut}) status following treatment with SHP099 for 72 hours. Graph was generated using data from Valencia-Sama *et al*., 2020. Error bars represent mean ± SD. *, $P<0.05$.
