# Supplementary material for: SHP2 Inhibition with TNO155 Increases Efficacy and Overcomes Resistance of ALK Inhibitors in Neuroblastoma
Source: Cancer Res Commun. 2023 Dec 27;3(12):2608–22. doi: 10.1158/2767-9764.CRC-23-0234 (PMC10752212; doi:10.1158/2767-9764.CRC-23-0234)
Supplement: Figure S2 — ALK-TKIs synergize with TNO155 in ALK mutant neuroblastoma cells. [file crc-23-0234-s06.pdf]

# Figure S2

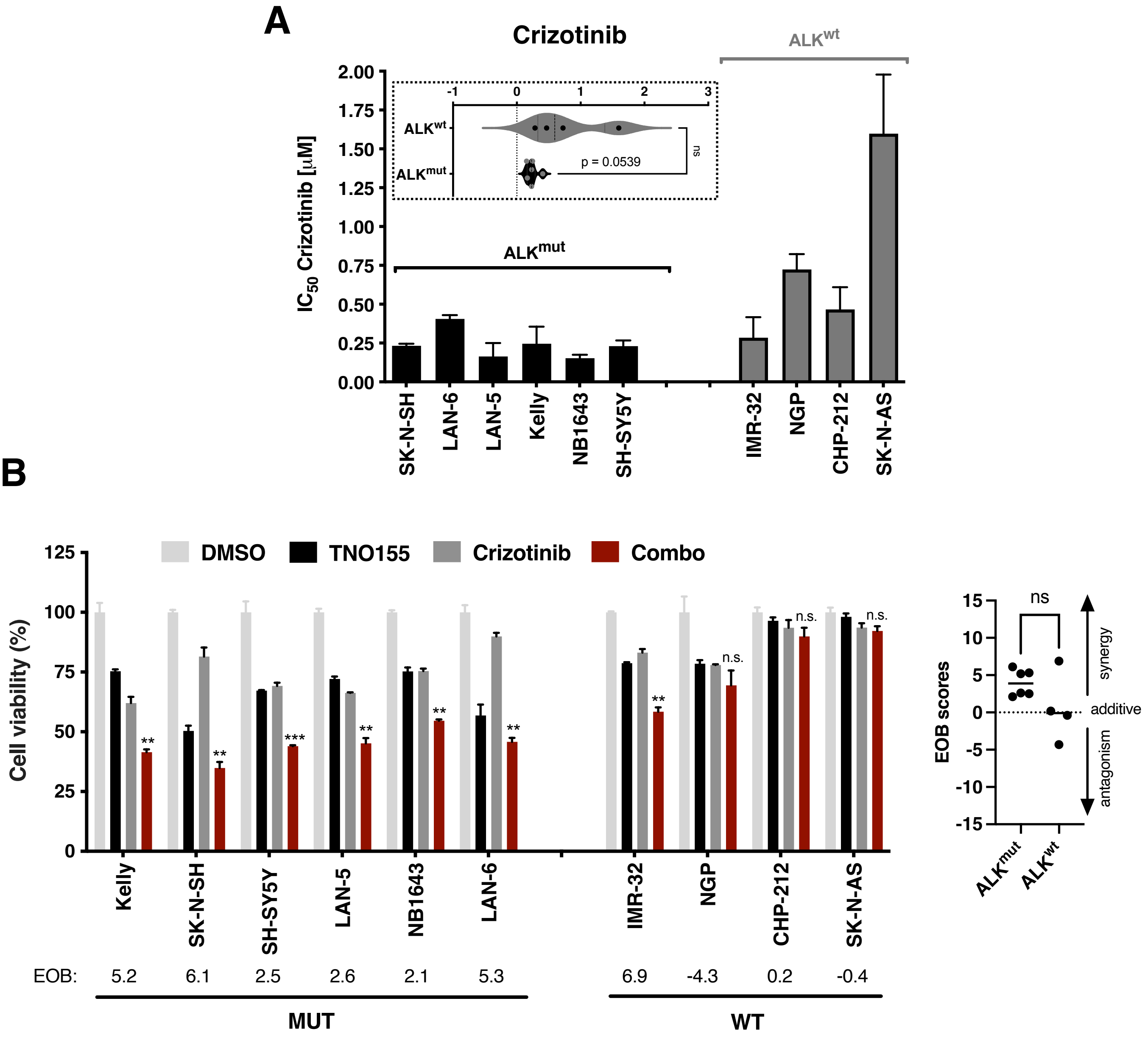
A
Crizotinib
ALKwt
ALKmut
IC50 Crizotinib [μM]
p = 0.0539
ns
SK-N-SH
LAN-6
LAN-5
Kelly
NB1643
SH-SY5Y
IMR-32
NGP
CHP-212
SK-N-AS
B
DMSO
TNO155
Crizotinib
Combo
Cell viability (%)
EOB: 5.2 6.1 2.5 2.6 2.1 5.3 6.9 -4.3 0.2 -0.4
MUT
WT
EOB scores
synergy
additive
antagonism
ns
n.s.

**Figure S2. ALK-TKIs synergize with TNO155 in ALK mutant neuroblastoma cells.**

**A**, Calculation of $IC_{50}$ in neuroblastoma cells with *ALK* wildtype ($ALK^{wt}$) or mutant ($ALK^{mut}$) status following treatment with Crizotinib for 72 hours. **B**, Cell viability (alamarBlue) analysis and determination of drug interaction in *ALK* wildtype (WT) or mutant (MUT) neuroblastoma cells treated with DMSO control, TNO155, crizotinib, or TNO155 plus crizotinib for 72 hours. Drug concentrations are shown in Supplementary Table S1A. Synergy was calculated using the Excess over Bliss (EOB) model. EOB scores > 0, synergistic. Error bars represents mean ± SD. **, P<0.01, ***, P<0.001, n.s., not significant.
