# Supplementary material for: SHP2 Inhibition with TNO155 Increases Efficacy and Overcomes Resistance of ALK Inhibitors in Neuroblastoma
Source: Cancer Res Commun. 2023 Dec 27;3(12):2608–22. doi: 10.1158/2767-9764.CRC-23-0234 (PMC10752212; doi:10.1158/2767-9764.CRC-23-0234)
Supplement: Figure S3 — TNO155 and ALK-TKI treatments increase apoptosis, reduce PI3K/AKT/mTOR signaling, and decrease growth of ALK mutant cells. [file crc-23-0234-s07.pdf]

Figure S3

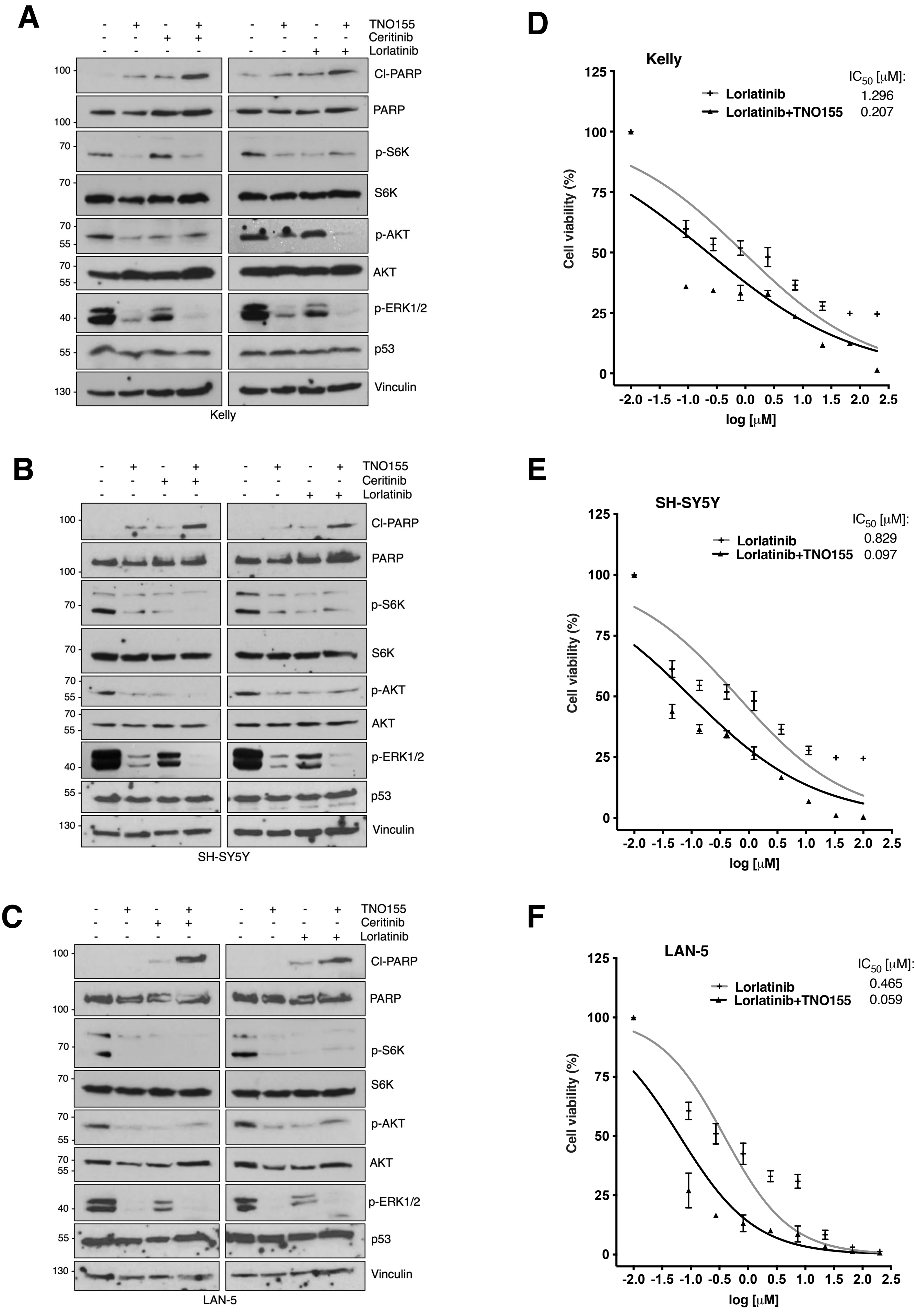
A
TNO155
Ceritinib
Lorlatinib
Cl-PARP
PARP
p-S6K
S6K
p-AKT
AKT
p-ERK1/2
p53
Vinculin
100
70
55
40
130
Kelly
B
SH-SY5Y
C
LAN-5
D
Kelly
IC50 [μM]:
Lorlatinib 1.296
Lorlatinib+TNO155 0.207
Cell viability (%)
log [μM]
E
SH-SY5Y
IC50 [μM]:
Lorlatinib 0.829
Lorlatinib+TNO155 0.097
F
LAN-5
IC50 [μM]:
Lorlatinib 0.465
Lorlatinib+TNO155 0.059
125
100
75
50
25
0
-2.0 -1.5 -1.0 -0.5 0.0 0.5 1.0 1.5 2.0 2.5

Figure S3. TNO155 and ALK-TKI treatments increase apoptosis, reduce PI3K/AKT/mTOR signaling, and decrease growth of ALK mutant cells.

A-C, Western immunoblots of Kelly (A), SH-SY5Y (B) and LAN-5 (C) cells treated with TNO155 [1μM], ceritinib [0.1μM] or combination (left panel), or TNO155 [1μM], lorlatinib [1 μM] or combination (right panel) for 24 hours. **D-F**, Cell viability (alamarBlue) and IC_{50} was assessed in Kelly (D), SH-SY5Y (E) and LAN-5 (F) cells treated with increasing concentrations (0 – 150 μM) of lorlatinib alone or lorlatinib plus TNO155 (0 – 150 μM) for 72 hours. Error bars represents mean ± SD.
