# Supplementary material for: SHP2 Inhibition with TNO155 Increases Efficacy and Overcomes Resistance of ALK Inhibitors in Neuroblastoma
Source: Cancer Res Commun. 2023 Dec 27;3(12):2608–22. doi: 10.1158/2767-9764.CRC-23-0234 (PMC10752212; doi:10.1158/2767-9764.CRC-23-0234)
Supplement: Figure S4 — SHP099 synergizes with ALK-TKIs in ALK mutant neuroblastoma cells. [file crc-23-0234-s08.pdf]

Figure S4

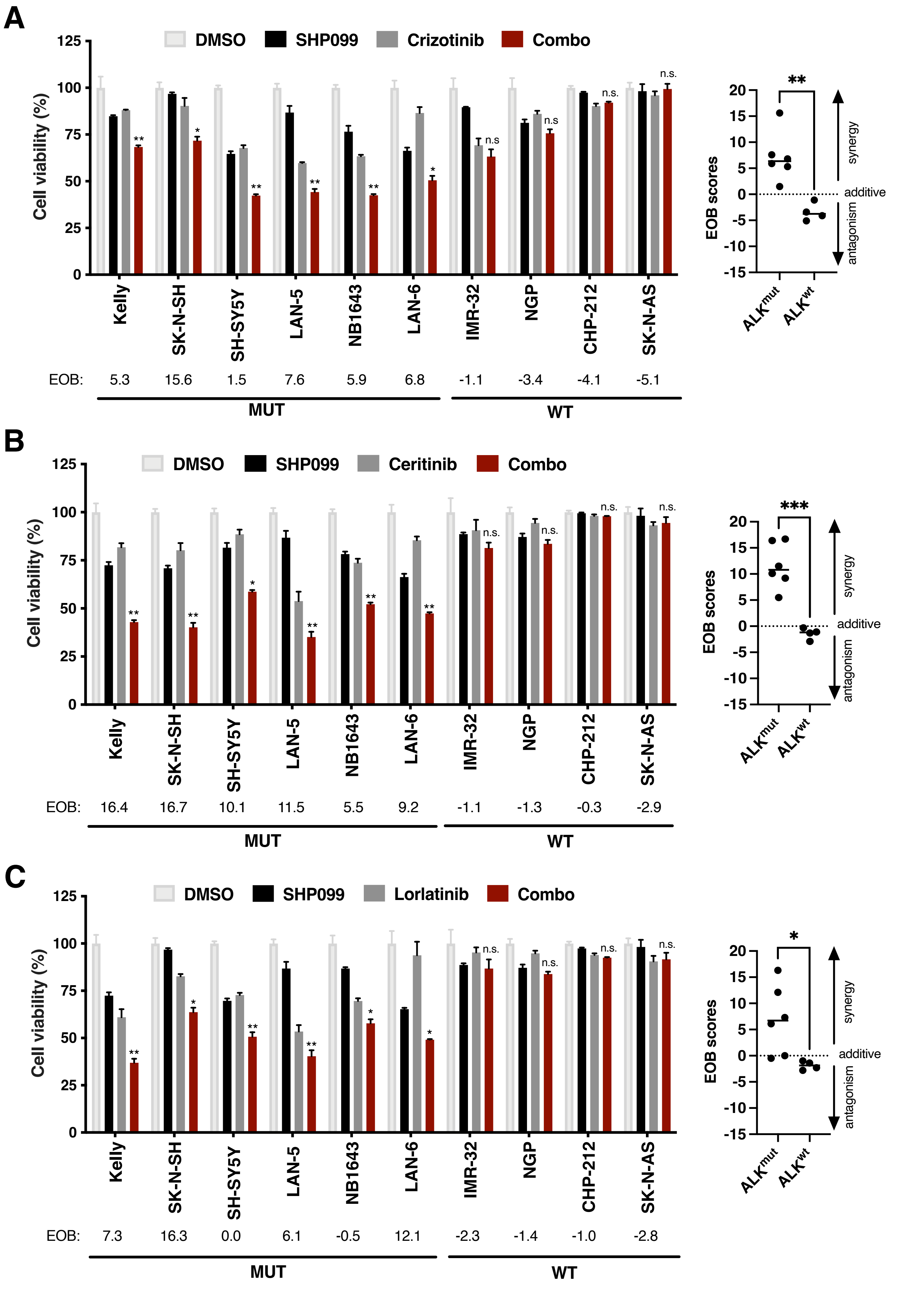
A
DMSO
SHP099
Crizotinib
Combo
Cell viability (%)
Kelly
SK-N-SH
SH-SY5Y
LAN-5
NB1643
LAN-6
IMR-32
NGP
CHP-212
SK-N-AS
EOB: 5.3 15.6 1.5 7.6 5.9 6.8 -1.1 -3.4 -4.1 -5.1
MUT
WT
EOB scores
synergy
additive
antagonism
ALK^mut
ALK^wt
B
DMSO
SHP099
Ceritinib
Combo
Cell viability (%)
EOB: 16.4 16.7 10.1 11.5 5.5 9.2 -1.1 -1.3 -0.3 -2.9
MUT
WT
EOB scores
synergy
additive
antagonism
C
DMSO
SHP099
Lorlatinib
Combo
Cell viability (%)
EOB: 7.3 16.3 0.0 6.1 -0.5 12.1 -2.3 -1.4 -1.0 -2.8
MUT
WT
EOB scores
synergy
additive
antagonism

Figure S4. SHP099 synergizes with ALK-TKIs in ALK mutant neuroblastoma cells.

A-C, Cell viability (alamarBlue) analysis and determination of drug interaction in neuroblastoma cells treated with DMSO control or SHP099 alone or in combination with crizotinib (A), ceritinib (B), or lorlatinib (C) for 72 hours. Drug concentrations are shown in Supplementary Table S1B. Synergy was calculated using the Excess over Bliss (EOB) model. EOB scores > 0, synergistic. Error bars represents mean ± SD. *, P<0.05, **, P<0.01, n.s., not significant.
