# Supplementary material for: SHP2 Inhibition with TNO155 Increases Efficacy and Overcomes Resistance of ALK Inhibitors in Neuroblastoma
Source: Cancer Res Commun. 2023 Dec 27;3(12):2608–22. doi: 10.1158/2767-9764.CRC-23-0234 (PMC10752212; doi:10.1158/2767-9764.CRC-23-0234)
Supplement: Figure S5 — High doses of SHP2 inhibitors sensitize ALK wildtype neuroblastoma cells to ALK-TKIs. [file crc-23-0234-s09.pdf]

Figure S5

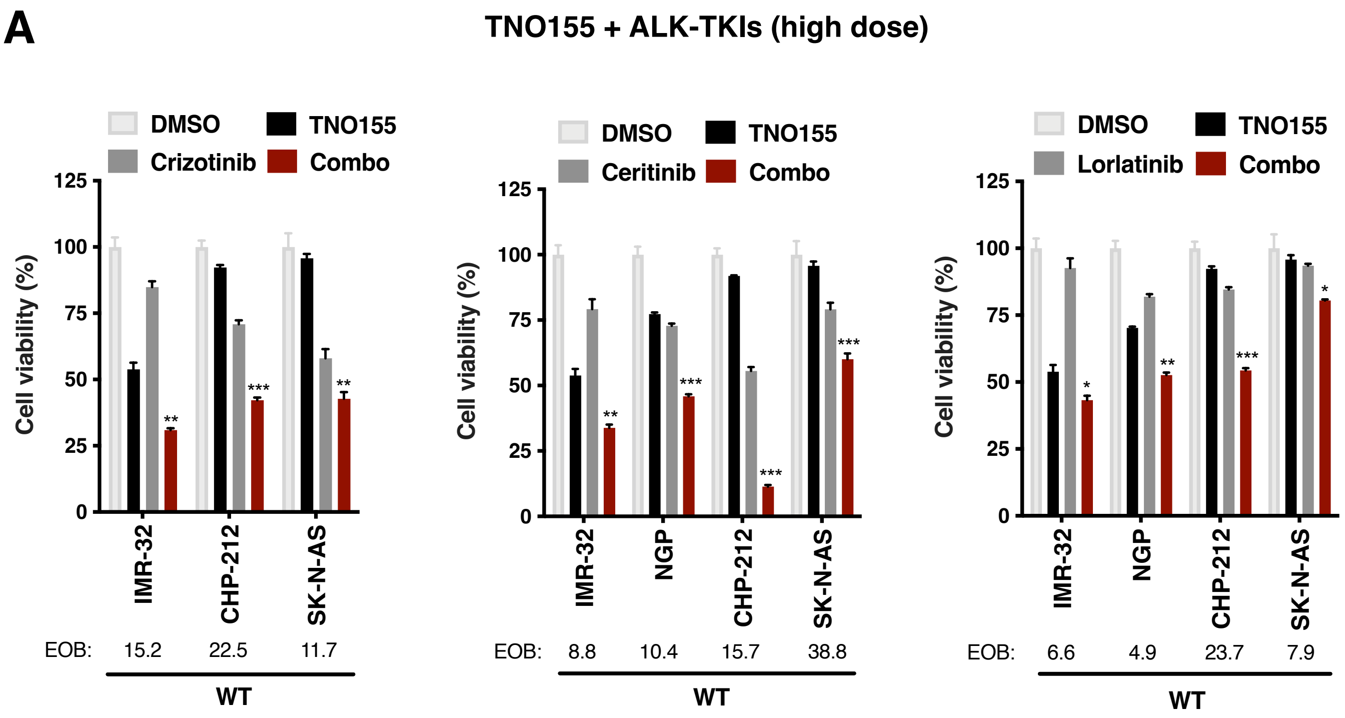
A
TNO155 + ALK-TKIs (high dose)
DMSO
TNO155
Crizotinib
Combo
Ceritinib
Lorlatinib
Cell viability (%)
IMR-32
NGP
CHP-212
SK-N-AS
EOB: 15.2 22.5 11.7
EOB: 8.8 10.4 15.7 38.8
EOB: 6.6 4.9 23.7 7.9
WT

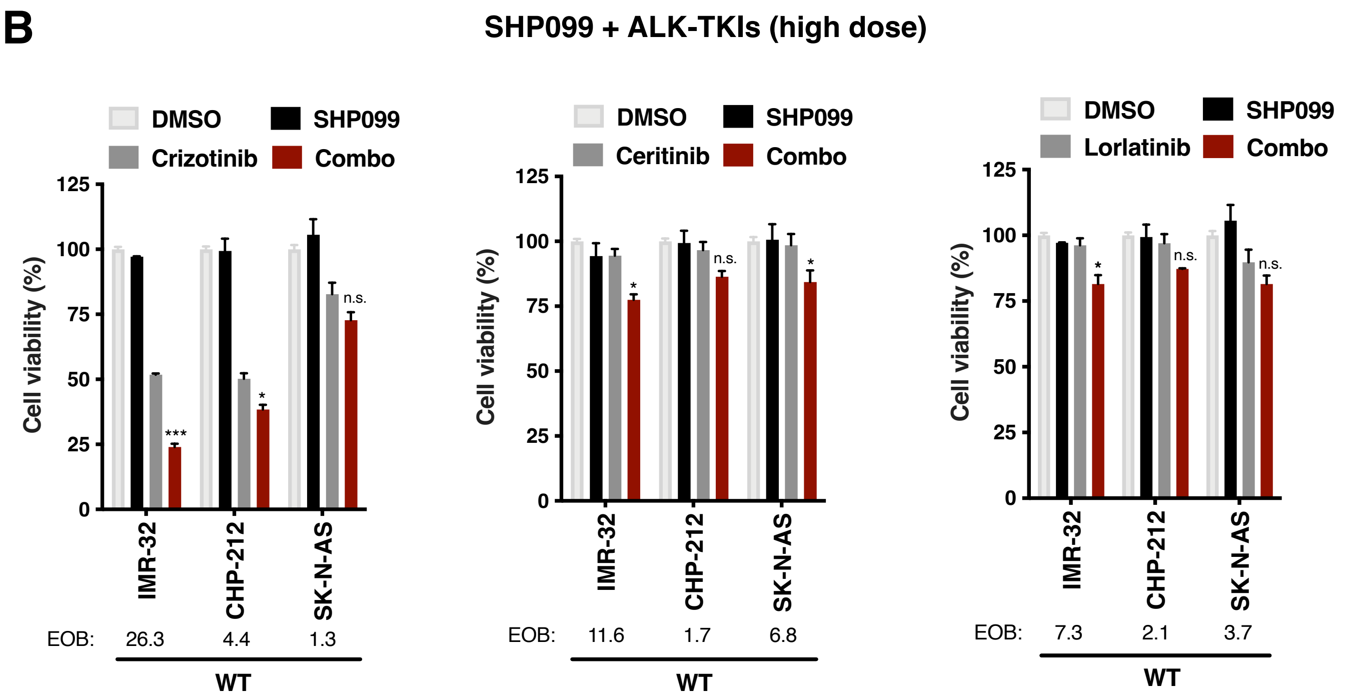
B
SHP099 + ALK-TKIs (high dose)
DMSO
SHP099
Crizotinib
Combo
Ceritinib
Lorlatinib
Cell viability (%)
IMR-32
CHP-212
SK-N-AS
n.s.
EOB: 26.3 4.4 1.3
EOB: 11.6 1.7 6.8
EOB: 7.3 2.1 3.7
WT

Figure S5. High doses of SHP2 inhibitors sensitize ALK wildtype neuroblastoma cells to ALK-TKIs.

A-B, Cell viability (alamarBlue) analysis and determination of drug interaction in neuroblastoma cells treated with TNO155 (A) or SHP099 (B) alone or in combination with crizotinib, ceritinib, or lorlatinib for 72 hours. Drug concentrations are shown in Supplementary Tables S1A and B. Synergy was calculated using the Excess over Bliss (EOB) model. EOB scores > 0, synergistic. Error bars represents mean ± SD. *, P<0.05, **, P<0.01, ***, P<0.001, n.s., not significant.
