# Supplementary material for: SHP2 Inhibition with TNO155 Increases Efficacy and Overcomes Resistance of ALK Inhibitors in Neuroblastoma
Source: Cancer Res Commun. 2023 Dec 27;3(12):2608–22. doi: 10.1158/2767-9764.CRC-23-0234 (PMC10752212; doi:10.1158/2767-9764.CRC-23-0234)
Supplement: Figure S6 — Assessment of TNO155 and ALK-TKI efficacy in zebrafish embryos. [file crc-23-0234-s10.pdf]

Figure S6

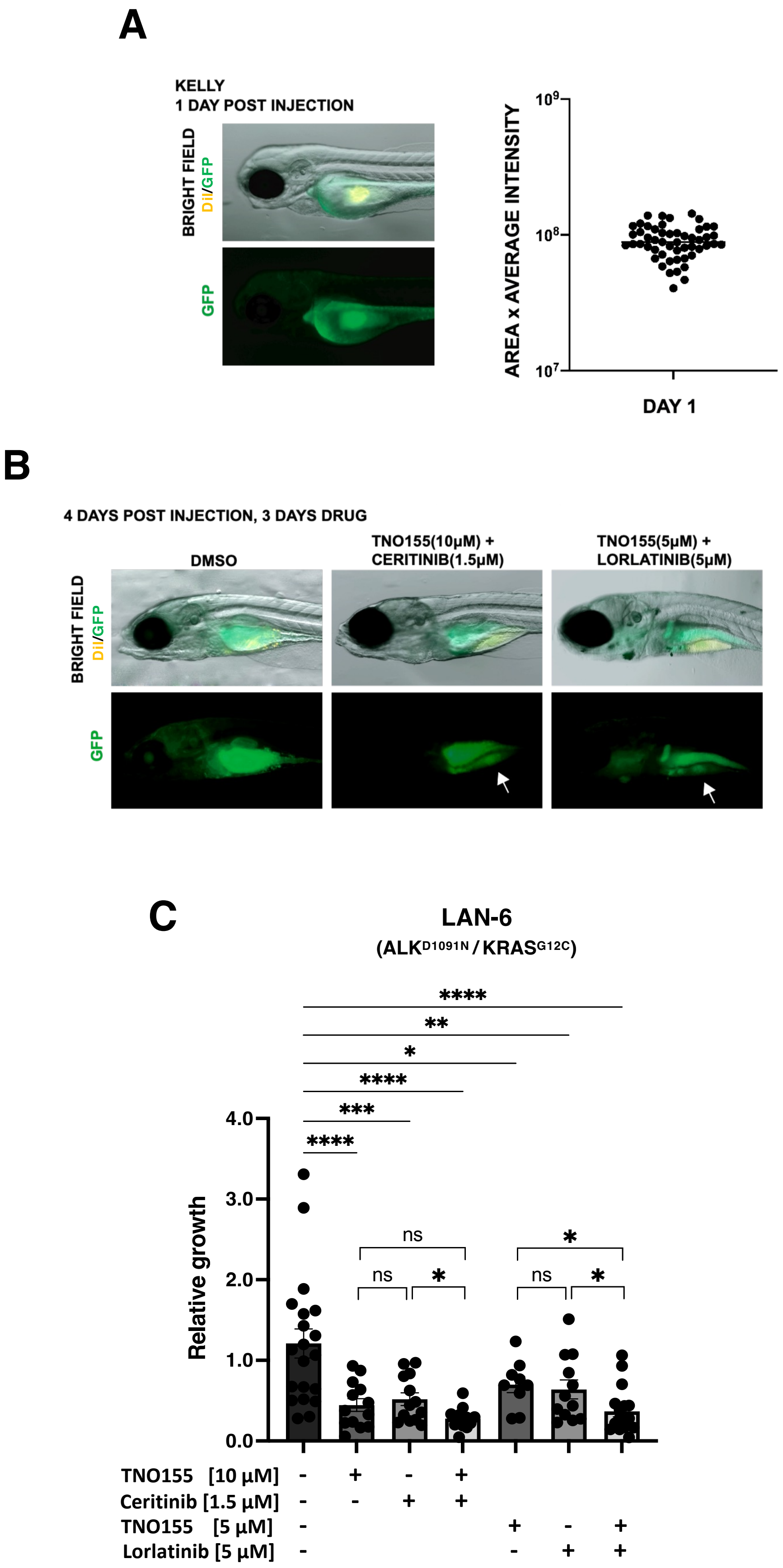

A
KELLY
1 DAY POST INJECTION
BRIGHT FIELD
DiI/GFP
GFP
AREA x AVERAGE INTENSITY
10^9
10^8
10^7
DAY 1
B
4 DAYS POST INJECTION, 3 DAYS DRUG
DMSO
TNO155(10μM) + CERITINIB(1.5μM)
TNO155(5μM) + LORLATINIB(5μM)
BRIGHT FIELD
DiI/GFP
GFP
C
LAN-6
(ALK^D1091N / KRAS^G12C)
****
**
*
****
***
****
ns
ns
*
*
ns
*
Relative growth
4.0
3.0
2.0
1.0
0.0
TNO155 [10 µM] - + - +
Ceritinib [1.5 µM] - - + +
TNO155 [5 µM] - + - +
Lorlatinib [5 µM] - - + +

Figure S6. Assessment of TNO155 and ALK-TKI efficacy in zebrafish embryos.

A-B, Larvae engrafted with GFP-expressing Kelly cells and co-stained with DiI dye were imaged and quantified by fluorescence (intensity per area) at 1 day post injection (dpi) (A) and submersed in TNO155 (5 or 10 μM), ceritinib (1.5 μM), lorlatinib (5 μM) alone or combination treatments at 35°C for 72 hours (B). **C**, GFP-expressing LAN-6 cells were submersed in TNO155 (5 or 10 μM), ceritinib (1.5 μM), lorlatinib (5 μM) alone or combination treatments at 35°C for 72 hours. GFP fluorescence (area x pixel intensity) was quantified and normalized to pre-treatment. Error bars represent mean ± SD. *, $P<0.05$, **, $P<0.01$, ***, $P<0.001$, ****, $P<0.0001$, ns, not significant.
