# Supplementary material for: SHP2 Inhibition with TNO155 Increases Efficacy and Overcomes Resistance of ALK Inhibitors in Neuroblastoma
Source: Cancer Res Commun. 2023 Dec 27;3(12):2608–22. doi: 10.1158/2767-9764.CRC-23-0234 (PMC10752212; doi:10.1158/2767-9764.CRC-23-0234)
Supplement: Figure S7 — Dual SHP2 and ALK inhibition reduces neuroblastoma cell migration. [file crc-23-0234-s11.pdf]

Figure S7

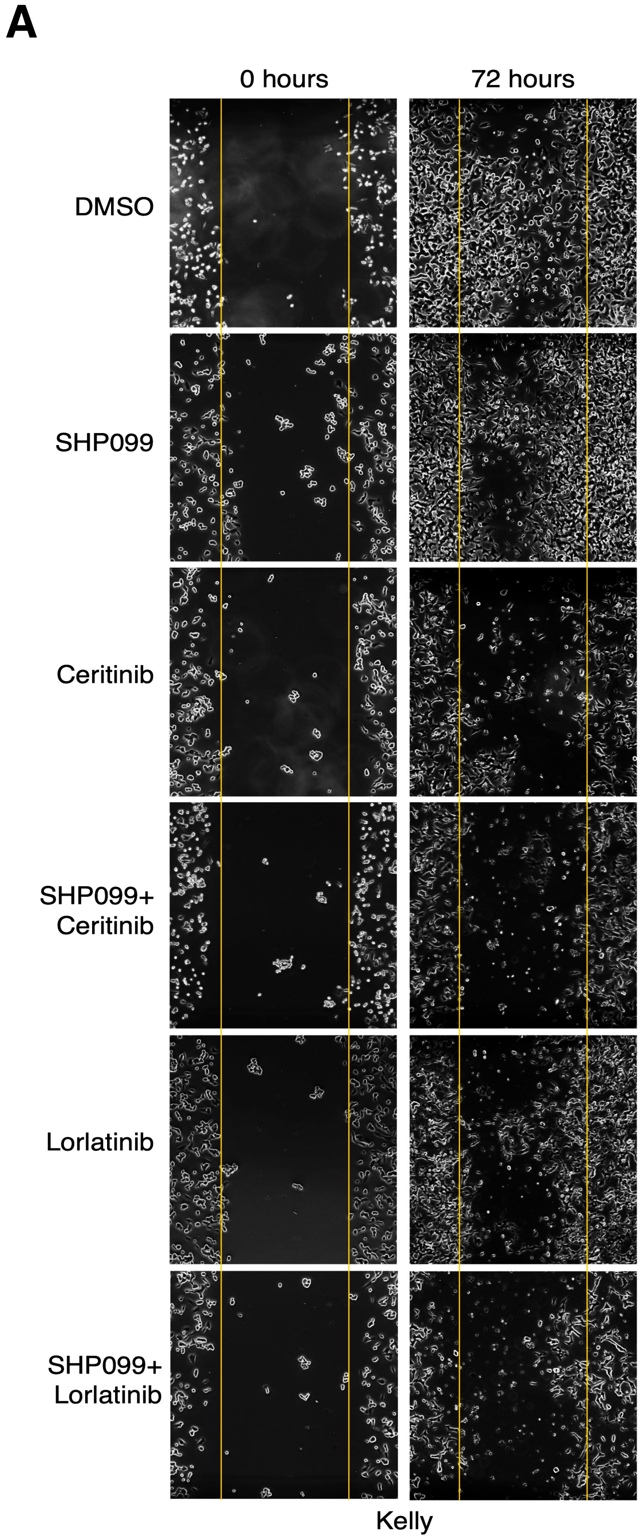
A
0 hours
72 hours
DMSO
SHP099
Ceritinib
SHP099+
Ceritinib
Lorlatinib
SHP099+
Lorlatinib
Kelly

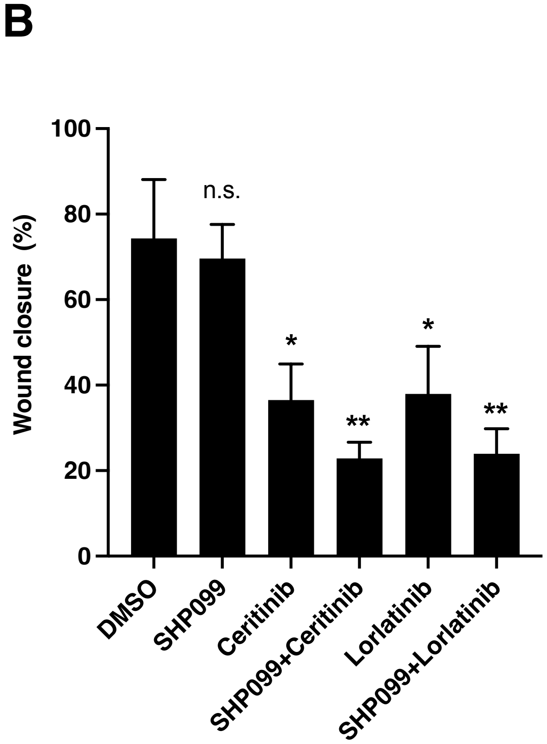
B
Wound closure (%)
100
80
60
40
20
0
n.s.
*
**
*
**
DMSO
SHP099
Ceritinib
SHP099+Ceritinib
Lorlatinib
SHP099+Lorlatinib

Figure S7. Dual SHP2 and ALK inhibition reduces neuroblastoma cell migration.

A-B, Scratch-wound assays were performed on confluent monolayers of Kelly cells treated with DMSO, SHP099 [30 μM], ceritinib [0.1 μM], lorlatinib [1 μM], or treatment combination for 72 hours. Representative images at 0 and 72 hours are shown (A). Graph demonstrates quantification of percent wound closure in cells treated with the indicated treatments (B). Error bars represent mean ± SD. *, $P<0.05$, **, $P<0.01$, n.s., not significant.
