# Supplementary material for: SHP2 Inhibition with TNO155 Increases Efficacy and Overcomes Resistance of ALK Inhibitors in Neuroblastoma
Source: Cancer Res Commun. 2023 Dec 27;3(12):2608–22. doi: 10.1158/2767-9764.CRC-23-0234 (PMC10752212; doi:10.1158/2767-9764.CRC-23-0234)
Supplement: Figure S8 — TNO155 plus Lorlatinib treatment decreases tumor burden in ALKF1174L murine xenografts. [file crc-23-0234-s12.pdf]

Figure S8

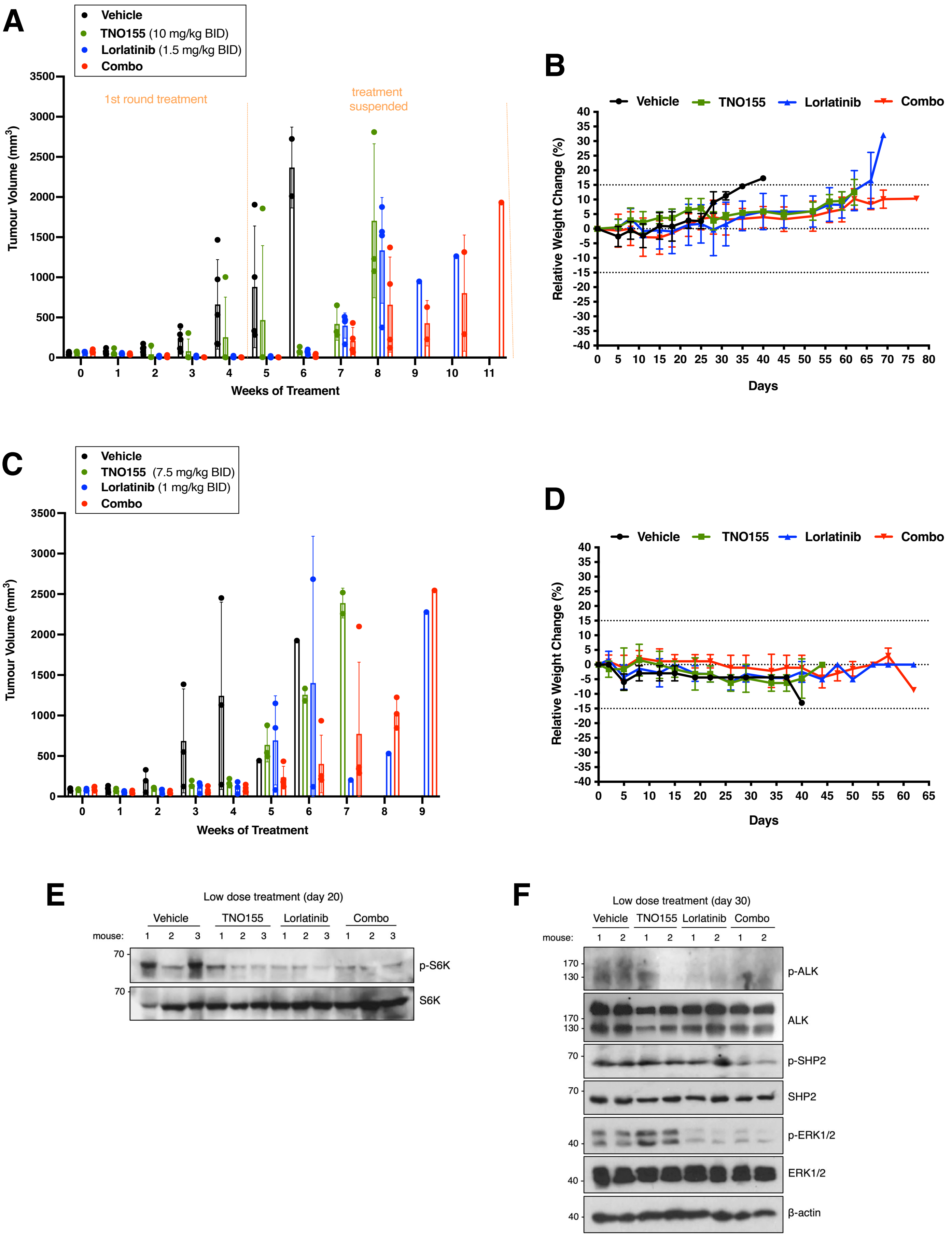

A
Vehicle
TNO155 (10 mg/kg BID)
Lorlatinib (1.5 mg/kg BID)
Combo
1st round treatment
treatment suspended
Tumour Volume (mm3)
Weeks of Treament
B
Vehicle
TNO155
Lorlatinib
Combo
Relative Weight Change (%)
Days
C
Vehicle
TNO155 (7.5 mg/kg BID)
Lorlatinib (1 mg/kg BID)
Combo
Tumour Volume (mm3)
Weeks of Treatment
D
Vehicle
TNO155
Lorlatinib
Combo
Relative Weight Change (%)
Days
E
Low dose treatment (day 20)
Vehicle
TNO155
Lorlatinib
Combo
mouse:
p-S6K
S6K
F
Low dose treatment (day 30)
Vehicle
TNO155
Lorlatinib
Combo
mouse:
p-ALK
ALK
p-SHP2
SHP2
p-ERK1/2
ERK1/2
β-actin

Figure S8. TNO155 plus Lorlatinib treatment decreases tumor burden in ALK^{F1174L} murine xenografts.

A-B, Kelly xenografts were treated by oral gavage with vehicle (n=5), TNO155 (10 mg/kg BID, n=4), lorlatinib (1.5 mg/kg BID, n=5), or combination (Combo, n=4) treatments for 4 weeks, and tumor volumes (A) and relative weight changes (B) were tracked until end-point. **C-D**, Kelly xenografts were treated by oral gavage with vehicle (n=3), TNO155 (7.5 mg/kg BID, n=3), lorlatinib (1 mg/kg BID, n=3), or combination (Combo, n=4) treatments for 9 weeks, and tumor volumes (C) and relative weight changes (D) were monitored until end-point. **E,** Protein expression analyses of tumor lysates treated with vehicle control (n=3), TNO155 (7.5 mg/kg BID, n=3), lorlatinib (1 mg/kg BID, n=3), or combination (Combo, n=3) treatments for 20 days. **F,** Protein expression analyses of tumor lysates treated with vehicle control (n=2), TNO155 (7.5 mg/kg BID, n=2), lorlatinib (1 mg/kg BID, n=2), or combination (Combo, n=2) treatments for 30 days.
