# Supplementary material for: SHP2 Inhibition with TNO155 Increases Efficacy and Overcomes Resistance of ALK Inhibitors in Neuroblastoma
Source: Cancer Res Commun. 2023 Dec 27;3(12):2608–22. doi: 10.1158/2767-9764.CRC-23-0234 (PMC10752212; doi:10.1158/2767-9764.CRC-23-0234)
Supplement: Figure S9 — TNO155 re-sensitizes lorlatinib-resistant ALKF1174L cells and tumors. [file crc-23-0234-s13.pdf]

Figure S9

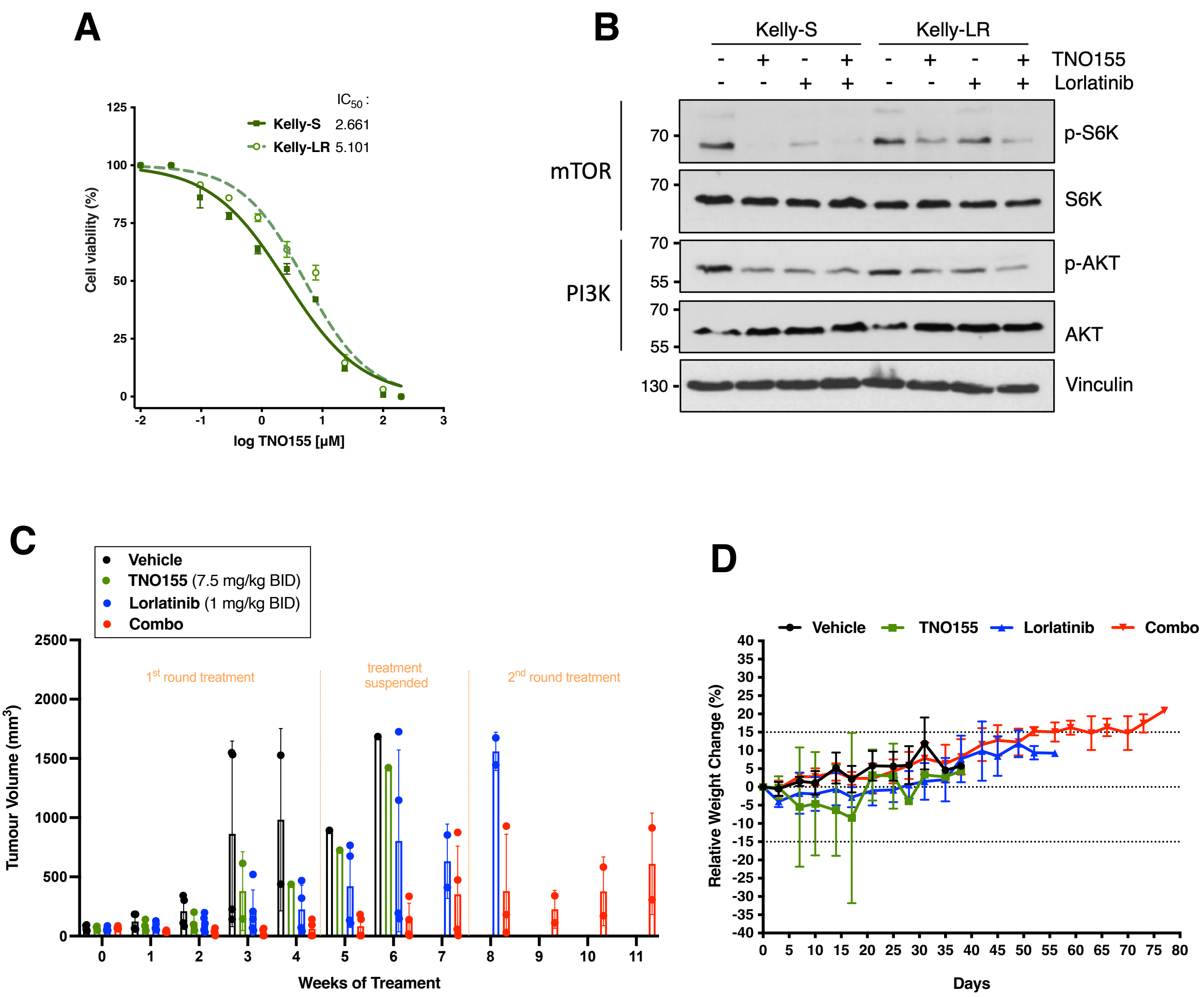

A
IC50 :
Kelly-S 2.661
Kelly-LR 5.101
Cell viability (%)
log TNO155 [μM]
B
Kelly-S
Kelly-LR
TNO155
Lorlatinib
mTOR
PI3K
p-S6K
S6K
p-AKT
AKT
Vinculin
C
Vehicle
TNO155 (7.5 mg/kg BID)
Lorlatinib (1 mg/kg BID)
Combo
1st round treatment
treatment suspended
2nd round treatment
Tumour Volume (mm3)
Weeks of Treament
D
Vehicle
TNO155
Lorlatinib
Combo
Relative Weight Change (%)
Days

Figure S9. TNO155 re-sensitizes lorlatinib-resistant ALK^{F1174L} cells and tumors.

A, Cell viability (alamarBlue) and IC_{50} was assessed in Kelly-S and Kelly-LR cells treated with increasing concentrations (0 – 150 μM) of TNO155 for 72 hours. **B**, Western immunoblots of Kelly-S and Kelly-LR cells treated with TNO155 [1.5 μM], lorlatinib [1 μM] or combination treatment for 6 hours. **C-D**, Kelly xenografts were treated by oral gavage with vehicle control (n=4), TNO155 (7.5 mg/kg BID, n=3), lorlatinib (1 mg/kg BID, n=5), or combination (Combo, n=4) treatments for 2 intervals (rounds) of 4 weeks each, with a treatment suspension break (3 weeks) in-between each round of treatment. Tumor volumes (C) and relative weight changes (D) were monitored until end-point. Death of one mouse treated with TNO155 (day 17) was attributed to causes unrelated to tumor burden or drug toxicity (cause of death: failure to awaken from anesthesia).
